# Large-scale interactive retrieval in art collections using multi-style feature aggregation

**DOI:** 10.1371/journal.pone.0259718

**Published:** 2021-11-24

**Authors:** Nikolai Ufer, Max Simon, Sabine Lang, Björn Ommer

**Affiliations:** Heidelberg Collaboratory for Image Processing, Interdisciplinary Center for Scientific Computing, Heidelberg University, Heidelberg, Germany; Universidad de Guadalajara, MEXICO

## Abstract

Finding objects and motifs across artworks is of great importance for art history as it helps to understand individual works and analyze relations between them. The advent of digitization has produced extensive digital art collections with many research opportunities. However, manual approaches are inadequate to handle this amount of data, and it requires appropriate computer-based methods to analyze them. This article presents a visual search algorithm and user interface to support art historians to find objects and motifs in extensive datasets. Artistic image collections are subject to significant domain shifts induced by large variations in styles, artistic media, and materials. This poses new challenges to most computer vision models which are trained on photographs. To alleviate this problem, we introduce a multi-style feature aggregation that projects images into the same distribution, leading to more accurate and style-invariant search results. Our retrieval system is based on a voting procedure combined with fast nearest-neighbor search and enables finding and localizing motifs within an extensive image collection in seconds. The presented approach significantly improves the state-of-the-art in terms of accuracy and search time on various datasets and applies to large and inhomogeneous collections. In addition to the search algorithm, we introduce a user interface that allows art historians to apply our algorithm in practice. The interface enables users to search for single regions, multiple regions regarding different connection types and holds an interactive feedback system to improve retrieval results further. With our methodological contribution and easy-to-use user interface, this work manifests further progress towards a computer-based analysis of visual art.

## Introduction

**Fig 1:**
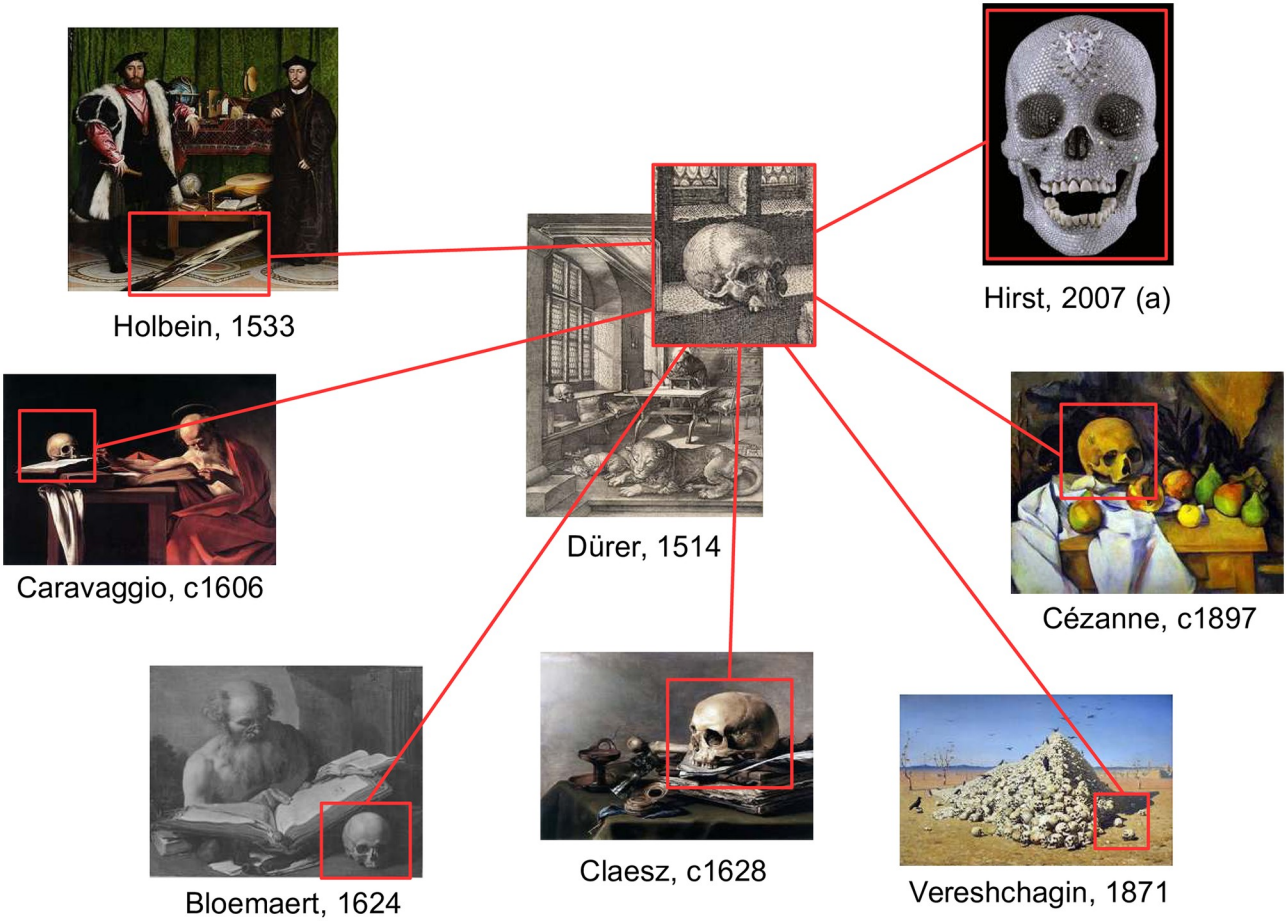
Example of contextual variations of a motif. Artists have painted the skull in different epochs, genres, and techniques. It appears, for example, in Hans Holbein’s The Ambassadors, Caravaggio’s painting of Saint Jerome, and still lives by Pieter Claesz or Modern artist Paul Cézanne. While it is always the same object, its symbolic function and meaning intended by the artist varies. For example, in Christian iconography, the skull relates to Saint Jerome, but more often, it refers to the concept of ‘memento mori’– a reminder of human mortality. Finding and tracking such motifs through time and space helps art historians to identify relations between artworks, the meaning and popularity of motifs, or how they have been adapted and sometimes altered in form and content. Image (a) is a slight modification of [4] licensed under CC BY 2.0. The remaining image material is shared by Wikimedia Commons [5] as public domain.

A central task of art history is to analyze the similarities between artworks to study reception processes, morphological variations of motifs or contextual changes in artworks, thus gaining more insight into artistic relationships. To investigate these visual similarities, art historians have to find the connection between specific image regions containing motifs or meaningful objects [1, 2]. This is particularly important for the study of iconographic questions, where researchers aim to determine and interpret specific themes, and pictorial symbols [3]. Fig 1 shows an example, which illustrates how different artists painted the motif of the skull throughout time but in varying contexts and styles. The skull is mostly linked to a religious image tradition, specifically to the figure of Saint Jerome and the concept of ‘memento mori’. In addition, in contemporary art the skull often appears highly commercialized–as exemplified by Damien Hirst’s skull-sculpture entitled ‘For the Love of God’ (2007), which is decorated with diamonds.

In recent years cultural institutions have digitized their collections, including paintings, graphics, drawings or illustrated books and thus created extensive digital image collections [6–13]. These digital holdings offer new research possibilities and enable art historians to analyze large collections of artworks, thus gaining new or complementary insights. Now that extensive data collections exist, we also need adequate methods to analyze them. Traditional methods, where artworks are manually examined, compared and categorized, are unfeasible. Imagine how long it would take to analyze tens of thousands of images by hand? To assist visual scholars, cultural institutions have implemented computer-based systems to accelerate and simplify this work. However, these systems often rely on metadata and allow only a text-based search [10–13]. This has many disadvantages because metadata has to be collected or generated for every image, which is an expensive process, requires expert knowledge and is prone to poor results due to incorrect information or mismatched labels. Another problem is that metadata does not encapsulate the diversity and visual characteristics of paintings that art historians are interested in. Therefore, we need efficient algorithms to search for visual similarities in large and diverse image collections based on pure visual input and user-defined queries. The following article addresses this problem and presents an efficient search algorithm and a user interface that allows users to select image regions containing objects or motifs of interest and find similar regions in a vast digital image collection.

One of the central problems in Computer Vision and, in particular, visual instance retrieval is how to measure the similarity between images or parts of images in a meaningful way. A direct comparison based on a pixel level is unreasonable because objects or motifs can undergo numerous changes. Therefore, abstract visual features or representations are extracted from images and encoded as numerical vectors. While Computer Vision has developed very efficient image representations for photographs [14, 15], they show severe deficiencies for artworks. Digitized art collections pose new challenges due to strong artistic abstractions and various motifs painted in different styles using changing artistic media and materials. Hence, previous image representations have to cope with a domain shift from photos to artwork and within art collections. Therefore, specifically tailored feature descriptors for the arts are necessary [16, 17]. These descriptors should be highly discriminative to find corresponding image regions and invariant to typical style variations at the same time. Therefore, we need feature descriptors that are especially suited for art [14, 15]. In the case of visual instance retrieval in the arts, two approaches are especially worth highlighting. Seguin et al. [18] used corresponding image pairs annotated by human experts to learn specifically tailored descriptors for the arts. However, such a supervised approach is very time-consuming and costly since it requires annotating thousands of pairs. In addition, the learned descriptors are only suitable for similar datasets, thus limiting their general applicability. An alternative method was proposed by Shen et al. [17] and is based on a self-supervised approach using spatial consistency to find corresponding image regions for training. While the results are very promising and expert annotations are no longer needed, the approach is only applicable on curated datasets with lots of repeating motifs for training. Furthermore, the automatic search for training image pairs is not feasible for large datasets since it scales quadratically with the dataset size. This article circumvents these drawbacks and introduces a multi-style feature aggregation that improves feature descriptors’ color and style-invariance without the need for strong supervision or specifically tailored datasets. For this, we use a feature extractor pre-trained on ImageNet [19] and a current style transfer model. We transfer each image into a fixed set of different styles, extract their features, and average the features over their stylizations to map them into the same distribution. In this way, we reduce their dependency in terms of style and significantly improve the search results across artworks.

For practical users, besides the existence of efficient search algorithms, it is equally important that these algorithms are made accessible in user-friendly interfaces. Both the commercial [20–22] and the academic [18, 23] side have made great efforts to develop such visual search interfaces. On the commercial side, image search is not new, and large technology companies, such as Google and Microsoft, made this feature already available in their search engines. However, their algorithms are built for photographs, and we observed experimentally that their search results are heavily influenced by color and style changes which is unsuitable for art historical use cases. Moreover, in their application, we observe that they provide only a holistic visual search either on entire or parts of images [20–22]. For example, in Bings’ selection search, the algorithm searches for entire images which are visually similar to the selected region. They are not able to find and localize regions within other images. See also the interface comparison in the Sect. Experiments. Another issue is that Googles’ and Microsofts’ image search are web search systems, where users cannot directly upload images, making it hard to apply them on their own image collections. On the academic side, there exist several search interfaces specifically designed for the arts [18, 23–25]. However, for most of them, the search is either restricted to holistic images [23–25], which makes it impossible to search for specific motifs or objects. Or they are designed for a particular dataset [18], which heavily limits their usability. In collaboration with art historians, we developed a user interface which was designed as an analytical tool for Art History. In this interface, users can upload their own dataset, select an image, and search for one or multiple regions across all images using the presented search algorithm. When searching for multiple regions, users can choose between different connection types. For this purpose, we introduce three scoring functions for evaluating different compositions and arrangements of retrieval regions using their individual retrieval scores. These include an OR, AND, and geometric score, where the geometric score also considers the consistency of geometric relationships between retrieval regions and query regions. Analyzing multiple regions across image collections enables art historians to follow compositions or motifs characterized by multiple parts, which is essential for iconographic questions. To improve the usability of the interface and to simplify the workflow of art historians, we have integrated several useful functionalities, such as a user and dataset access management, searching through metadata, naming searches, browsing through old search results, marking favorite results or switching between different visualizations of the retrievals.

### Contributions

This article presents a visual search algorithm and user interface to find semantically similar or identical image regions in extensive art collections. It allows art historians to identify visual patterns or relations between artists and study the development of motifs and topics throughout the history of art. This article is an extension of a workshop presentation [26] of this work, where we have presented the basic retrieval algorithm. Our main contributions are summarized as follows:

1. We present a feature aggregation strategy for the visual search in art collections, which successfully reduces the domain gap and improves overall retrieval results.
2. The introduced iterative voting combined with efficient nearest-neighbor search enables finding and localizing small motifs in an extensive dataset within seconds.
3. We demonstrate that the proposed method significantly outperforms the state-of-the-art in terms of retrieval time and accuracy for searching across artworks. We provide an in-depth analysis of the algorithm that goes far beyond the previous presentation.
4. We newly present a user interface that allows art historians to use our visual search algorithm in practice. In addition to searching for single regions, it allows searching for multiple regions regarding different connection types, and contains an interactive feedback system to refine search results further.
5. To demonstrate the usability and implications of our search interface, we newly present a case study on an art historical research question.

The visual search interface is accessible on our project website, see the URL in the data availability statement. With our easy-to-use visual search interface and methodological contribution, this work marks further progress towards the machine-supported analysis of art.

## Related work

In recent years, several researchers in computer vision have focused on the development and application of computer-based methods for the arts. Our work is embedded in this context, and in the following section, we present existing research and highlight our contribution to related work.

### Computer vision in the arts

Computer vision and art history share fundamental questions since both are concerned with visual perception and investigate the characteristics of images to understand their content. Past years have shown an increase of fruitful and successful collaborations between scholars from computer vision and art history, demonstrating the willingness, interest, and necessity of interdisciplinary research. Collaborations have included the analysis of artworks utilizing computer-based methods [27–29] or the development of novel algorithms in order to meet art historical needs and answer new research questions [30]. With the breakthrough of generative models, new applications came up, like transferring artistic styles onto images [31–33] or creating some kind of computer-generated art [34, 35]. Encouraged by the general success of computer vision for analyzing large image collections, researchers have transferred algorithms for classification and object recognition from real photos to artworks to solve various tasks. This includes finding iconographic elements, gestures, or objects in artistic images [29, 36–40], or classifying artworks according to their style, genre, or artist [9, 41–44]. Besides finding objects and classifying paintings, art historians are interested in visual similarities and patterns between artworks to gain new knowledge about artistic relations or the meaning and development of motifs. To solve this task without manual effort, researchers proposed algorithms to find visual similarities in art collections automatically [17, 45, 46]. While these approaches show promising results, they are limited to small collections since they require a pairwise comparison of all images and thus, scale quadratically with the dataset size. Our work also aims to assist art historians with finding visual connections between artworks, but in contrast to previous works, we focus on a large-scale search of visual queries selected by the user. This makes it possible to analyze large art collections by performing several targeted searches for motifs that interest the user.

### Visual instance retrieval

Visual instance retrieval is one of the fundamental tasks in Computer Vision. It has attracted considerable attention in the last decades due to the rapid increase in stored and processed image material. It deals with finding images based on a visual query. It is a challenging problem due to the semantic and intention gap, which describes the need to have a higher level of abstraction from low-level pixel data and the difference between the user’s search intent and the provided query. Numerous techniques have been developed for this task with successful classical [27, 47], as well as deep learning-based [48–50] methods. Compared to object detection with successful algorithms such as FastRCNN [51] and YOLO [52], the particular challenge in visual instance retrieval is that there are neither predefined object categories nor a large amount of training data. The visual search queries are arbitrary image regions containing arbitrary motifs or objects. Previous methods for visual instance retrieval used hand-crafted feature point descriptors like SIFT [53] in a bag-of-words manner [27, 47, 54]. Convolutional neural networks (CNNs) significantly improved visual instance retrieval [55]. But the main research in this area is focused on photos showing the same place [56, 57] or object [58]. However, this is unsuitable for art history because the field studies very heterogeneous image collections with large domain shifts induced by variations in styles, artistic media, and material. Researchers who tackled searching across art collections often focused on image level-based approaches using a whole image as query [59–63]. However, such a holistic approach does not allow searching for individual motifs or objects in images, which is a central requirement of art historians. The most similar approaches to our work that allow a regional image search and focus on art datasets are the works of Seguin et al. [16] and Shen et al. [17]. However, the approach of Seguin et al. [16] is based on strong supervision in such a way that hundreds of images had to be classified by an art historian for one month and thus, cannot be applied to new datasets without a great deal of expert labor. Shen et al. [17] circumvents the need for labeled data and uses a self-supervised approach by mining corresponding image regions to learn more style-invariant features, which is similar to [64, 65]. However, this self-supervised approach is limited to curated datasets with a huge set of re-occurring motifs, and their sliding-window approach for searching for a single query takes several hours on a large dataset. In contrast to previous works, our method reduces the domain-induced differences across artistic images and improves retrieval results without the need for strong supervision or curated image collections. Furthermore, our voting-based retrieval algorithm enables scholars to find and localize motifs and objects at various scales and locations across large and heterogeneous datasets within seconds.

### Visual search interfaces

Most search functionalities in digital image archives are still text-based searches through metadata [10–13, 25], which are limited since they require metadata and cannot capture the visual variety of images. In the following, we focus on visual search based systems. Several companies, such as Google, Microsoft, and Idée Inc., offer engines to search the web for images [20–22]. However, these algorithms are not designed for artwork, and most of the interfaces do not allow to upload images to search through own datasets directly. Only TinEye [22] offers a fee-based API for this purpose, but their service is restricted to find exact and altered copies and can not retrieve semantically similar motifs. Furthermore, we experimentally observed that these engines provide only a holistic image search. This is also the case for Bings’ selection search. Although the user can select an image and mark an area to search for, the retrievals are entire images similar to the selected region. Finding and localizing the selected region within other images is not possible. See also the qualitative interface comparison in the Sect. Experiments. On the academic side, with the emergence of new visual search algorithms for the arts, significant effort has been made to provide user interfaces for their practical application. For example, the Oxford Painting Search [23] is based on the work of Crowley et al. [27, 66] and is a search tool for the ARTUK dataset [7]. It allows searching for text queries, color, textures, object categories, and holistic images. However, their interface is restricted to a specific dataset and does not support searching on a regional level. Ahmed Elgammal et al. created the fee-based service ArtPI [24], a search tool for museums and auction houses. The system enables text and visual searches for holistic images. Besides visual content, users might also search for similar paintings regarding color or light treatment. However, their service also does not allow the search for single motifs or objects within images, which is essential for art history. In 2018, the Digital Humanities Laboratory of the École Polytechnique fédérale de Lausanne presented Replica [18]. In their interface, users can search for text, holistic images, or image regions in the digitized art collection of the Giorgio Cini Foundation in Venice, which mainly includes artworks from the Italian Renaissance. It provides different viewing modes and allows user feedback to improve results. Their interface is most similar to ours. However, their search algorithm is based on expert annotations of related images and cannot be applied to other datasets without great effort. Moreover, although they allow searching for regions within images, their first filtering step is based on a holistic image search [16], which allows their algorithm to work on such a large dataset. However, this brings the risk that small motifs, which are not well covered by global features, are overlooked in the first step and will not be final search results. The most significant disadvantage of all previously mentioned systems is that they either allow only a holistic image search [20–24] or are limited to specific art collections [18], which dramatically limits their practical application. In contrast, our interface allows users to search for entire images, single and multiple image regions, define spatial relations for regions, upload own collections, promote user feedback to refine results, search through metadata, browse through old searches and visualize results with different viewing modes. These functions ensure that the presented interface is a useful research tool for art historians.

## User interface

To enable art historians to apply our search algorithm in practice, we additionally developed a user interface. It was established in cooperation with art historians to meet their particular requirements. The interface allows users to upload and analyze individual image collections, where the algorithm retrieves not only identical but also similar semantic regions. This is important to reconstruct reception processes or investigate morphological variations of motifs over space and time. Besides searching for a single query region, the interface allows users to search for multiple regions, where they can choose between different connection types. This includes a geometric connection, where the algorithm returns images where the arrangement of retrieved regions are geometrically consistent with the query regions. Thereby, users can explicitly specify the weighting between the visual similarity against the geometric consistency before starting the search. This specifies how strong the algorithm should down weight geometric deviations in terms of pairwise distances and angles between the arrangement of retrieved compared to query regions. For a detailed explanation of all connection types, see the Sect. Retrieval algorithm. This multi-region search enables art historians to find similar regions and examine compositions or motifs characterized by multiple parts, which is essential for iconographical studies. The interface with a demonstration video is available on our project website, see the URL in the data availability statement. In the following, we describe the interface architecture, workflow, and additional functionalities in more detail.

### Architecture

**Fig 2:**
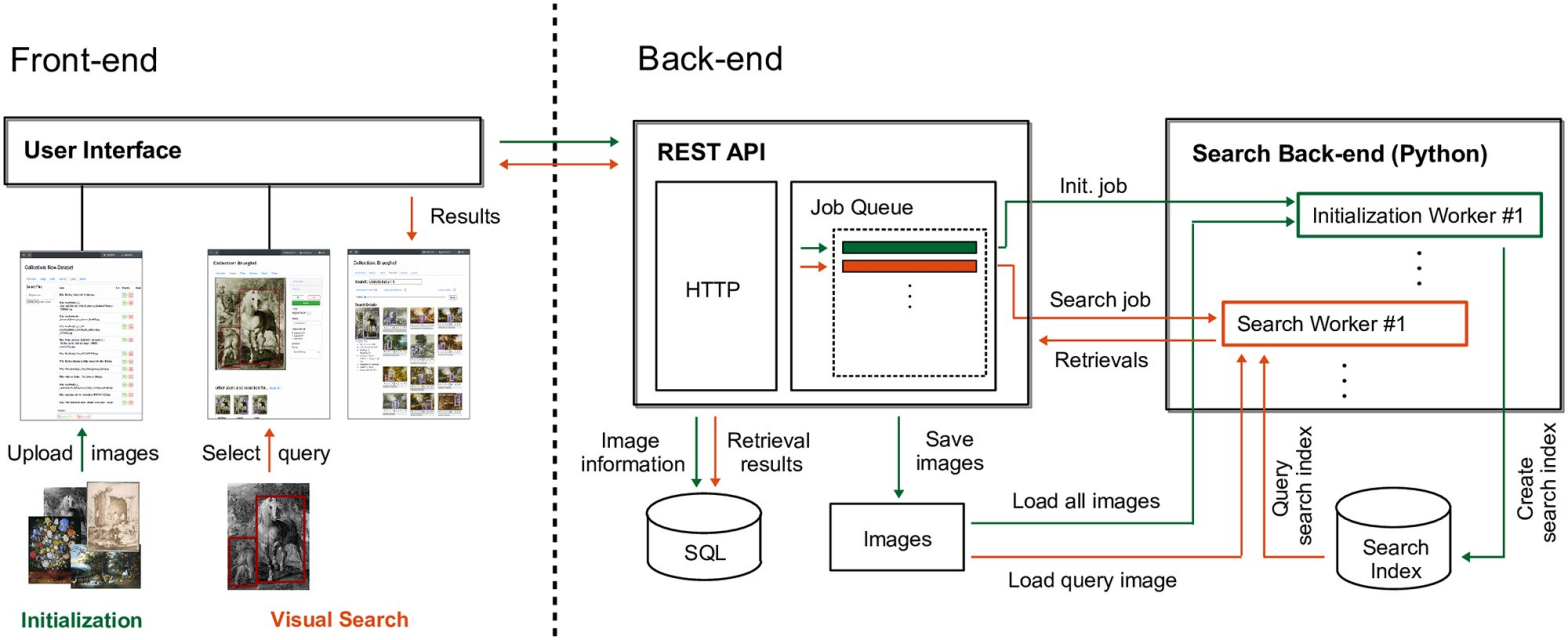
Overview of the interface architecture. The application is divided into a front-end and back-end, where the front-end is served as a single-page application and represents the interface for the user. The back-end consists of a REST API and the search back-end, where the search back-end consists of multiple initialization and search workers. The image material is shared by Wikimedia Commons [5] as public domain.

Following modern web development standards, the user interface is divided into a front-end and a back-end. The front-end was developed using the Angular framework [67] and is served as a single-page application. The back-end consists of a REST API (webserver) and the search back-end, where the REST API manages the access to images and metadata and administrates the search back-end. The search back-end consists of multiple initialization and search workers running in the background, which are responsible for the actual initialization and search across the selected image collection. See Fig 2 for an overview.

Splitting the back-end into an API and a search back-end allows for asynchronous initializations and search requests. The search back-end can run on different machines because the communication with the webserver is managed through the API interface. For parallelizing single user requests and efficiently searching through multiple datasets, the search back-end consists of multiple concurrently running workers that can perform different initialization and search requests in parallel. The webserver maintains a job queue which is read by the workers. If a worker gets a job on an unknown dataset, it first has to load the respective search index into working memory (CPU mode) or onto the GPU (GPU mode). Since an index can have several tens of GB in size, this can take up to several minutes. Then after loading, the usual search speed is achieved without additional overhead. When a worker has loaded a specific search index, it only performs search requests for this dataset for a fixed time after the last search. After this time, the worker accepts requests concerning other datasets so that the old search index is potentially replaced with a new one. This limits the number of required workers and the amount of memory used. Overall, running multiple search workers allows us to dynamically manage searches across multiple datasets without loading the index for each individual search. After the search, all retrievals, user feedback, and refinements are stored in a SQL database. This allows a comfortable navigation through favorites and old search results and hence a convenient analysis of previous searches and visual relations within the image collections.

Orthogonal to our approach with concurrent workers is the idea of design patterns similar to MapReduce [68]. In this design pattern, several workers are running simultaneously on different machines, where each worker is responsible for searching through a subset of the dataset. After the partial searches, the results are combined to a final result. This allows distributing the search over different machines and upscaling the searchable dataset size almost arbitrarily with the number of machines. Since our application is targeted at cultural institutions, where typically no computer cluster is available, we did not integrate such a design pattern into our interface. However, we see this as a potential future work if a computer cluster is available for this task and much larger datasets are targeted.

### Workflow

**Fig 3:**
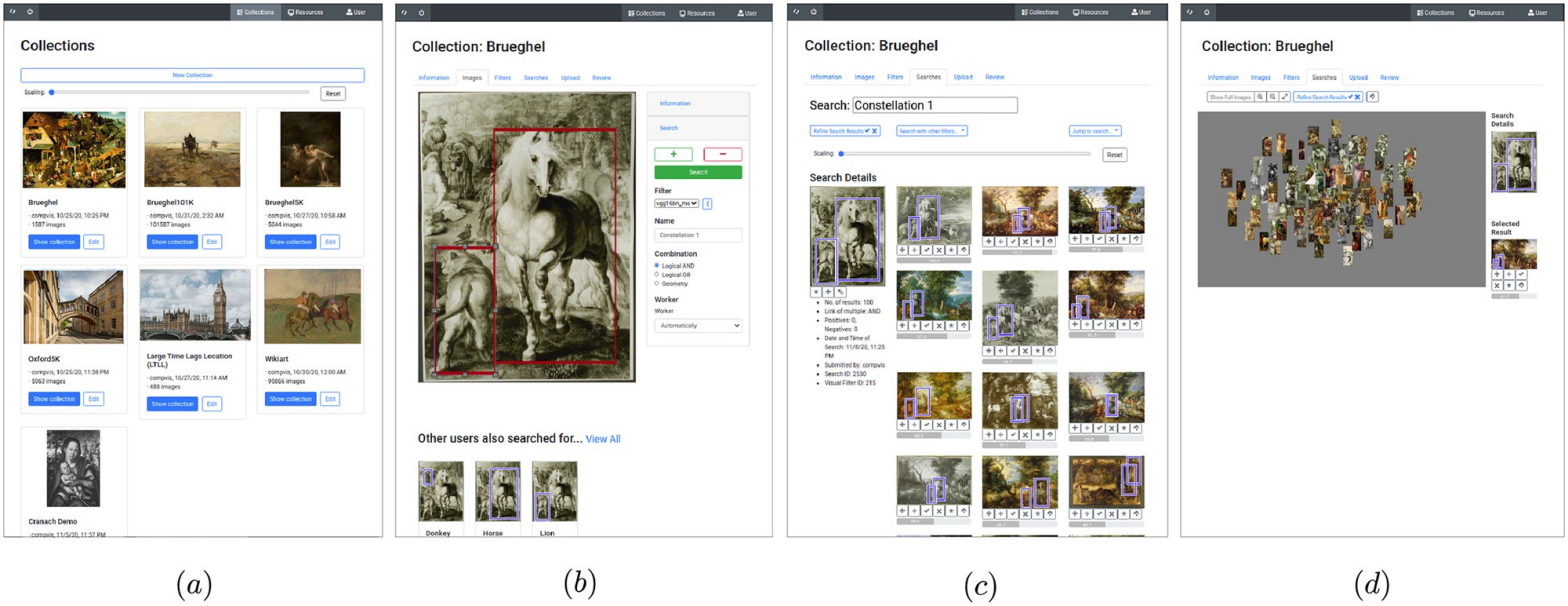
Visualization of the interface workflow. First, the dataset to be searched is selected, or a new dataset is created and initialized (a). Then an image can be chosen, and one or multiple search boxes can be selected and searched across the dataset (b). The retrievals are displayed in an ordered list (c) or with a two-dimensional t-SNE embedding [69] (d). Image material is shared by Wikimedia Commons [5] either as public domain or under a CC0 license.

The workflow of the interface is as follows. First, the user selects an image collection or uploads a new one and starts the initialization process. During this offline preparation stage, the algorithm extracts the features and builds the search index. This offline stage has to be done only once for each dataset. Afterwards, the index is stored and loaded if needed. In the online search stage, users can mark one or multiple regions of interest by drawing bounding boxes and start the search process for these regions across the whole dataset. If users search for multiple regions, they can specify the connection type, and in the case of geometric scoring how strong the algorithm should take the geometric relationship between regions into account. If the desired geometric connectivity is set very low, the algorithm searches for images in which the selected regions occur somewhere in the image. And if it is set very high, the algorithm searches for the exact relative positioning of the selected regions, where small deviations are heavily penalized in the retrieval ranking. After the search algorithm is terminated, the results are visualized in the order of decreasing similarity. An overview of the workflow with the interface at different stages can be found in Fig 3. The mentioned aspects of the retrieval algorithm are explained in detail in the Sect. Retrieval system.

Additional functions add to the interface’s usability. It contains a user management where users with passwords can be created and assigned different access rights for the individual datasets. Users can upload or manually enter additional metadata to image collections, such as the artist name, the style or the year of origin. The metadata is stored in a database and allows to find specific images for the visual search via keyword search. All visual searches with retrieval results are stored in a database, and users can name their searches and mark retrieval favorites. This allows to quickly find old searches and analyze datasets based on previous search results. The retrieval results can be displayed in different ways. Either in a list with descending retrieval score or in a two-dimensional embedding that arranges retrievals according to their visual similarities. For the embedding, we extract the features of the retrieved regions, calculate their mutual similarities, and compute their two-dimensional embedding using t-SNE [69]. Finally, we shift and normalize the coordinates by subtracting the query embedding coordinate and dividing by the largest extension in x- and y-direction. Thus, the query region is located in the center of the embedding, and the retrievals are arranged around it according to their pairwise visual similarities. The layout of the interface supports easy and intuitive navigation through the search process. Each function aims to simplify the workflow for art historians. For example, the simultaneous view of selected favorites allows for comparative analysis, and a close-up view of retrieved regions enables a focused study of objects. In addition, the two-dimensional embedding of search results gives a quick overview and the possibility of finding patterns within search results faster.

## Retrieval system

This section describes the retrieval system in detail. It consists of a novel image representation and an iterative voting-based retrieval algorithm. The image representations for encoding visual content is central for visual instance retrieval since it determines the similarity between image regions. We introduce an image representation specifically tailored for searching through digital art collections. It is based on a novel multi-style feature aggregation that reduces domain-specific differences and improves overall retrieval results. The actual retrieval algorithm consists of an iterative voting procedure, which allows to find and localize small motifs across an extensive dataset within seconds. In addition, the retrieval system includes a multi-region search that allows art historians to search for compositions of motifs and a user feedback system to improve the retrieval results further. In the following, we describe the multi-style feature aggregation and then the iterative voting-based retrieval algorithm in detail.

### Multi-style feature aggregation

Significant domain shifts induced by differences in styles and techniques are a major challenge for image representations in art collections. Features from models trained on photos perform well when searching for similar regions across photographs. However, they show severe deficiencies in art collections because they are strongly biased by the inherent visual variations in artistic images. In our approach, we try to alleviate this issue by exploiting current style transformation models to project all images into the same distribution and reduce their domain-specific differences. For this, we stylize all images into a fixed set of styles using a set of style templates from the dataset, extract their features using an ImageNet pre-trained CNN and finally aggregate those to obtain a single image representation. The resulting representation is more invariant to colors and styles compared to the initial representation and thus simplifies the retrieval task.

**Fig 4:**
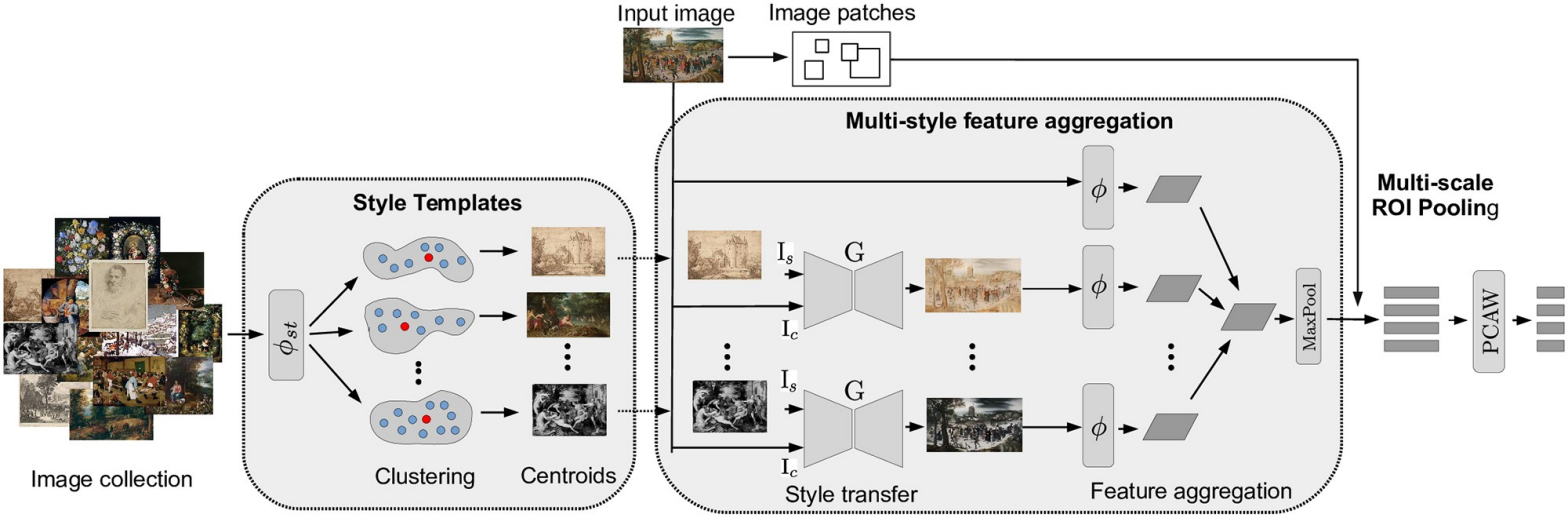
Overview of the multi-style feature aggregation. It consists of three main parts: First, we extract the features of all images using a style classification network, cluster them in the feature space, and select the cluster centers as style templates. Second, given an input image, it is stylized concerning all style templates using a universal style transfer model. Then, all stylized image features are extracted and aggregated to the final multi-style feature representation. Third, given the feature map of this representation and a set of image patches, their feature descriptors are extracted using multi-scale ROI pooling. Please see the text for more details. Image material is shared by Wikimedia Commons [5] either as public domain or under a CC0 license.

Our approaches consist of the following three steps, which are also visualized in Fig 4. First, we determine a fixed set of images in different styles within the dataset, which serve as our style templates. Second, we use a universal style transfer model to project each image into all styles given by the style templates, extract the features of the stylizations and aggregate them into a single image representation. Third, we use a region-of-interest pooling in a multi-scale fashion to extract feature descriptors for image regions needed in the search phase. In the following, we describe each step in more detail.

#### Selection of style templates

First, we select several images from the dataset as our style templates which will be subsequently used for the stylization. To get more robust image representations, we select images with rather diverse styles. To find such a set, we first need an image representation that is discriminative concerning artistic styles. For this, we train a style classification network in a supervised fashion to identify 27 different styles on the Wikiart dataset [6]. The resulting network $\phi_{st}:I\to R^{m}$ maps images into an *m*-dimensional feature space. We project all images into this feature space and group them into *k*_*s*_ clusters using K-Means. After the clustering, we select the images which are closest to the cluster centers in the feature space as our style templates $S=\{I_{s}|1\leq s\leq k_{s}\}$. This selection is sufficient because the style templates do not have to represent the variation of all styles within the dataset but provide a subset of diverse styles that we can use to average. In our experiments, we set *k*_*s*_ = 3 since it has provided the best retrieval performance. See the ablation study in the Sect. Experiments.

#### Feature aggregation of image stylizations

The multi-style feature aggregation module consists of an universal style transformation model *G* and a feature encoder *ϕ*. The universal style transformation model G:I×I→I takes two images *I*_*c*_, *I*_*s*_ as input and generates an image *G*(*I*_*c*_, *I*_*s*_). Thereby, the new image *G*(*I*_*c*_, *I*_*s*_) represents a stylization of *I*_*c*_ with the content from *I*_*c*_ and the style from *I*_*s*_. It is based on the network architecture of Li et al. [70] and was pre-trained on the MS-COCO dataset [71]. The feature encoder *ϕ* maps images into a *c*-dimensional feature space, where the output is a feature map with the spatial resolution of the input image divided by a fixed stride and the channel depth *c*. In our implementation, we use a VGG-16 with batch normalization [72], pre-trained on ImageNet [19], and truncated after the fourth block. Now, given an input image *I* and a set of style templates S, we compute all its stylizations *G*(*I*, *I*_*s*_), extract their feature maps *ϕ*(*G*(*I*, *I*_*s*_)) and fuse them into a single feature map $\phi_{ms}\left(I,S\right)$. For this, we considered three aggregation strategies. First, our default approach, where we average over all stylizations by

$$\begin{array}{c} {\left(\phi_{ms}^{mean}(I,S)\right)}_{ijc}:=\text{mean}({\left(\phi (I)\right)}_{ijc},{\left(\phi (G(I,I_{1}))\right)}_{ijc},\ldots ,{\left(\phi (G(I,I_{k_{s}}))\right)}_{ijc}), \end{array}$$ (1)

where *i*, *j* are the spatial coordinates in *x*−, *y*-direction, and *c* the channel coordinate. As alternative strategies, we also considered taking the element-wise maximum over the stylizations by

$$\begin{array}{c} {\left(\phi_{ms}^{max}(I,S)\right)}_{ijc}:=\text{max}({\left(\phi (I)\right)}_{ijc},{\left(\phi (G(I,I_{1}))\right)}_{ijc},\ldots ,{\left(\phi (G(I,I_{k_{s}}))\right)}_{ijc}), \end{array}$$ (2)

and concatenating all style templates along their channel dimensions via

$$\begin{array}{c} {\left(\phi_{ms}^{concat}(I,S)\right)}_{ij}:=[{\left(\phi (I)\right)}_{ij1},\ldots ,{\left(\phi (G(I,I_{1}))\right)}_{ij1},\ldots {\left(\phi (G(I,I_{k_{s}}))\right)}_{ijk_{s}}], \end{array}$$ (3)

which has the disadvantage that the channel depth and thus the size of the feature map increases significantly. For all aggregation strategies, we also included the feature map of the original image. This has proven advantageous since some fine-grained information useful for the retrieval task is lost during the style transformation. The ablation study comparing the different aggregation strategies has shown that averaging is the most efficient method, see the Experiments section.

#### Multi-scale region of interest pooling

The voting-based retrieval algorithm relies on encoding each image with a set of local patches, where the nearest neighbors of patches within the query vote for the final retrieval bounding boxes. How the retrieval system works and which regions we use will be explained in the next section. However, to search for nearest neighbors of image regions with different sizes, it is necessary to encode them with feature descriptors of a fixed length. Therefore, we apply Precise ROI Pooling [73] on the aggregated feature map $\phi_{ms}\left(I,S\right)$ and compress their feature dimension using principal component analysis with whitening (PCAW). The use of principle component analysis is beneficial since it suppresses non-specific information and directs the search to the most discriminative dimensions in the feature space. To be as stable as possible against scale changes, we extend the standard region-of-interest pooling in a multi-scale fashion. For standard region-of-interest pooling, the feature map is extracted on a single scale which is used for the ROI pooling and all regions independent of their size. In contrast to this, we extract the aggregated feature maps in seven different scales and select the most appropriate scale for each region by choosing the smallest feature map with a higher resolution than the respective pooling grid of the region. This significantly improves the accuracy for finding corresponding regions over different scales, see also the respective ablation study in the Sect Experiments.

### Iterative voting-based retrieval algorithm

To enable art historians to find specific objects or motifs within digital art collections, they must be able to select specific image regions and find them in other images of the dataset. This is a challenging requirement since encoding images with a single feature descriptor cannot capture arbitrary regions, and encoding all possible regions with various positions, sizes, and aspect ratios is unfeasible. Furthermore, limiting these regions to a few proposals is also difficult since this requires knowing in advance what the user is actually interested in. To tackle this issue, we introduce an iterative voting-based on a set of generic local patch descriptors. We encode each image using a moderate number of quadratic patches on multiple scales. Then, given a query region, we select the most discriminative patches within the query region and search for their nearest-neighbors across the whole dataset. Each of these nearest neighbors of local query patches votes for a retrieval bounding box, and multiple votes in an image are aggregated to the final retrieval bounding box. Thereby, the predicted retrieval bounding boxes can have any position and size with the aspect ratio of the query region and differ from the local patches used for the voting. Our approach allows finding and localizing small motifs with a moderate number of local patches per image, leading to more reliable search results by combining several weak search queries. In addition to the basic algorithm, we introduce two extensions that are useful in practice. To allow art historians to study the composition of motifs, we extend the algorithm with a multi-region search regarding different connection types, including their mutual geometric relationship. This is based on the single region search in combination with different scoring functions to evaluate all possible configurations. To improve search results further, we also integrated an interactive user feedback system, where the retrievals can be refined in a subsequent search using retrievals marked as correct or incorrect.

#### General structure

We visualize the general structure of the retrieval algorithm in Fig 1, where individual parts are marked and tagged. The retrieval algorithm consists of an offline preparation and an online search stage. The offline preparation stage has to run once before the actual search to build the search index for the nearest neighbor search. Therefore, the feature descriptors of all local patches are extracted using the multi-style feature network (F1), which are then stored in the search index for fast nearest neighbor search (F2). In the online search stage, users select a query region, most discriminative local patches inside this region are extracted (N1), and then our voting is applied to their k-nearest-neighbors, which results in localized retrieval bounding boxes (N2). Finally, through local query expansion and re-voting, the retrieval results are further improved (N3). Users can give feedback on these results, marking them as correct and incorrect, and restart the search to refine the retrievals further. Besides the single region search, users can mark multiple regions as a query with different scoring functions. In the following, we describe each part of the retrieval algorithm in detail.

#### Image encoding with local patch descriptors (F1)

**Fig 5:**
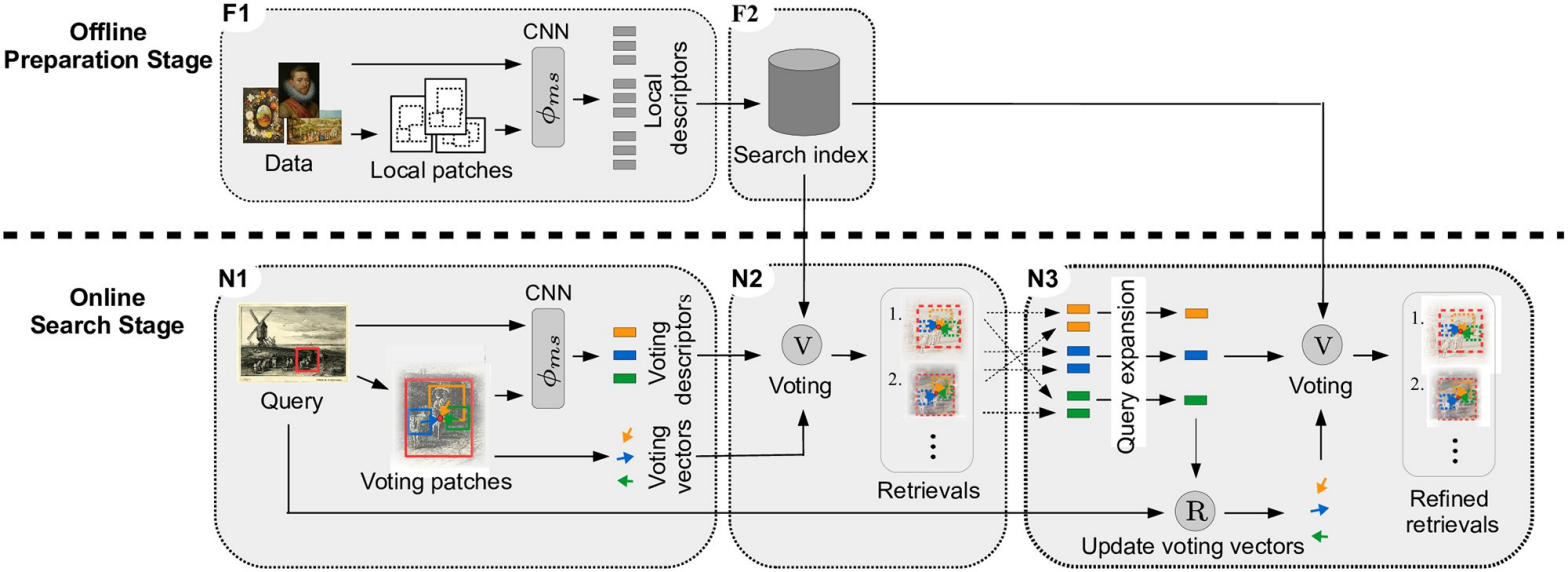
General structure of the retrieval algorithm. The retrieval algorithm consists of an offline preparation and an online search stage. During the offline preparation stage, features of local patches on multiple-scales are extracted for all images (F1). Then, they are compressed and stored in the search index (F2). During the online search stage, descriptors of discriminative local patches within the query region are extracted (N1). Their k-nearest neighbors across the whole dataset are determined, and our voting procedure aggregates multiple local matches to retrieval bounding boxes (N2). Finally, the results are refined using local query expansion and re-voting (N3). Please, see the text for more details. Image material is shared by Wikimedia Commons [5] either as public domain or under a CC0 license.

Our strategy for encoding images with local patch descriptors consists of two steps, see also F1 in Fig 5. First, we generate a set of quadratic patches on multiple scales for each image in a sliding window manner. We start with a patch size and stride of 1/12 and 1/50 regarding the largest image side and increase them over 6 scales with 2 scales per octave for the patch size and 3 scales per octave for the stride up to a patch size and stride of 1/2 and 1/16, respectively. Second, to filter more discriminative patches we apply non-maximum suppression on the feature activations, which we obtain by summing over the feature channel of the ROI pooled multi-style feature maps. Thereby, we limit the maximal number of patches per image to 4000 and additionally include the entire image in this set. By this, we cover each image with a set of more discriminative quadratic patches on multiple scales. We extract their feature descriptors using the multi-style feature aggregation and thus represent each image *I* with a set of local patch descriptors D(I), which are subsequently used in the voting procedure. To keep the search index more compact and allow searching in larger datasets, we reduce the maximal number of local patches by 375 for each additional 20K images in the dataset. The optimal parameter choice depends on the dataset properties and available computational resources. We have chosen the parameters by experimental validation on our benchmarks. See also the corresponding ablation study in the Sect. Experiments. Please note that the previously described patches are only used for the voting, and the final retrieval bounding boxes differ from those.

#### Search index and geometric database (F2)

All local patch descriptors for all images D are extracted and organized in a search index for fast nearest neighbor search, see also F2 in Fig 5. For the index, we use Product Quantization (PQ) for approximate nearest neighbor (ANN) search with Inverted File Index (IVF) [47, 74], where we resort to the GPU implementation of [74]. In addition, we store the corresponding names and positions of the local patches in an array to be able to assign retrieved search index ids to local patches in the dataset. For the search based on PQ, the descriptors are divided into slices on which individual codebooks with centroids are computed. Then, each descriptor is represented as a sequence of centroids, and distances between descriptors are approximated by the sum of centroid distances, which are previously computed and stored in a lookup table. This allows fast ANN search because after determining the centroids of the query, the distances can directly be computed using the lookup table. For the PQ algorithm, we utilize 96 sub-quantizers with 8 bits allocated for each sub-quantizer. The IVF algorithm is used to limit the search to the most promising local patch descriptors. Therefore, all descriptors are grouped into clusters with respective centroids using K-Means. Then the distances from a query to all centroids are used to limit the search to the closest clusters. We generated 1024 clusters with K-Means in our implementation, using 30 for the nearest neighbor search. Before the search index can be populated with descriptors, the PQ codebooks and the IVF clustering must be learned. We do this for each dataset separately using local patch descriptors from 1000 randomly selected images of the respective dataset. This concludes the offline preparation stage.

#### Query formulation with local patch descriptors (N1)

After the search index has been initialized, search requests can be made. When users select an image and mark a rectangle *q* as the query region, the first step is the query formulation based on the query region itself and additional local patch descriptors, see also N1 in Fig 5. Therefore, we extract local patches as described in the section about image encoding and select the most discriminative among them in two steps. First, we remove all patches with too few overlap and much smaller than the query region. On the remaining patches, we apply non-maximum suppression based on their feature activation and select up to 25 patches with the highest activation. Afterwards, we extract the features of the query region and the selected local query patches, which we denote D(I,q) and which serve as our voting descriptors. In addition to the descriptors, we also store the voting vectors, i.e. the offset from the center of the local patches to the center of the query region and the relation between the diagonals of the query region and local patches.

#### Voting based on local matches (N2)

**Fig 6:**
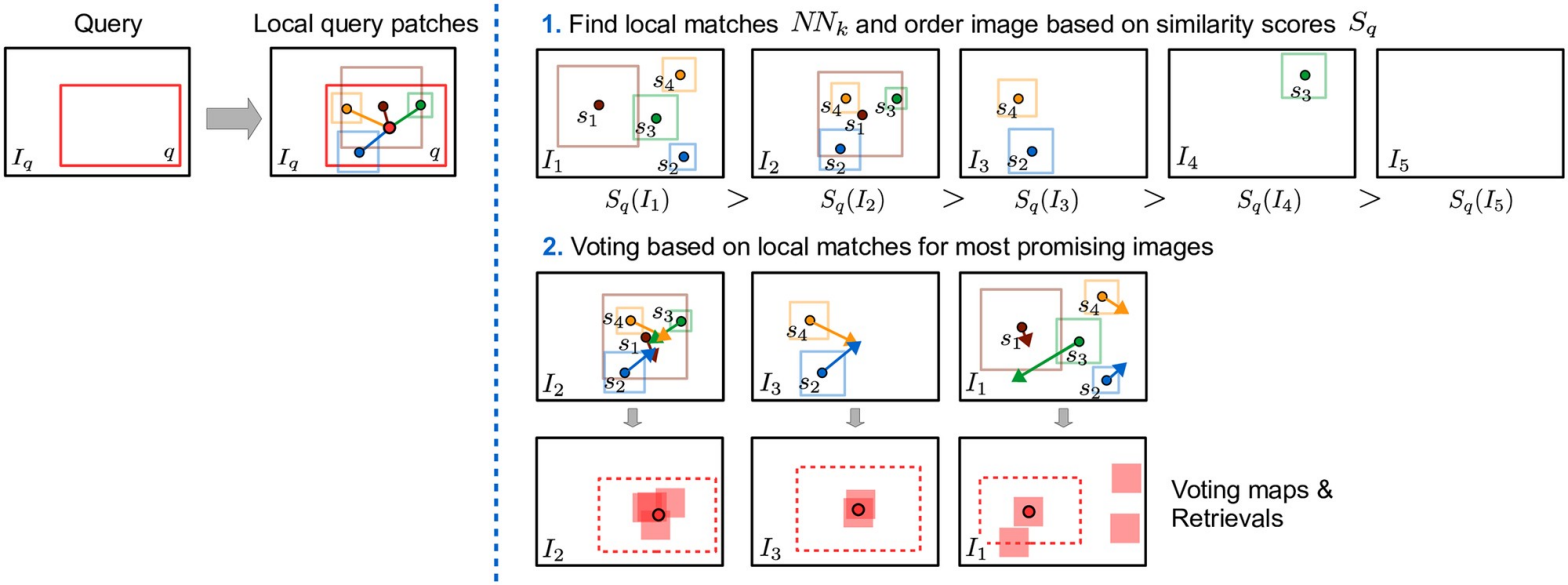
Overview of our voting based on local matches. The voting consists of two main steps. First, for each local query patch, we search for its k-nearest neighbors across the dataset, order all images based on their sum of local matching scores, and focus on the images with the highest score. Second, each local match votes for a specific center and scale of the retrieval bounding box, which are aggregated to the final retrieval results.

To recapitulate, each image *I* in the dataset is encoded with a set of local patch descriptors D(I) on multiple scales. All local patch descriptors of all images D are stored in the search index. And for a given query region *q* in image *I*_*q*_, it’s descriptor and additional local query patch descriptors $D(I_{q},q)$ within the query region are extracted. Then, the voting consists of two main steps. First, we search for the k-nearest neighbors of all local query patch descriptors and perform majority-based voting to filter most promising images. Second, all local matches in the filtered images vote for retrieval bounding boxes, which are fused to final retrieval results. The two steps are visualized in N2 of Figs 5 or 6 in more detail, and will be explained in the following.

In the first step, for each *f* ∈ *D*(*I*_*q*_, *q*) we search for its k-nearest neighbours $N\;N_{k}\left(f,D\right)$ across all local patch descriptors D, where we set *k* = 2048 and call (*f*, *g*) a local match for any *g* ∈ *NN*_*k*_(*f*, *D*). For all local matches (*f*, *g*), we define their local matching scores by

$$\begin{array}{c} s_{f}\left(g\right)=\text{exp}(-\frac{{∥g-f∥}_{2}^{2}\;}{\;∥\hat{g}{-f∥}_{2}^{2}}), \end{array}$$ (4)

where $\hat{g}\in N\;N_{k}\left(f,D\right)$ with a fixed rank of 512 and works as a reference distance. To be able to limit the subsequent voting to a subset of most promising images, we define the similarity of query region *q* to an image *I* by summing over all local matches in *I*, i.e.

$$\begin{array}{c} S_{q}\left(I\right)=\sum_{f\in D(I_{q},q)}\text{max}(\{s_{f}\left(g\right)|\;g\in N\;N_{k}\left(f,D\right)\cap D\left(I\right)\}), \end{array}$$ (5)

where we consider at most one hit per image. Based on *S*_*q*_, we select the 500 most promising images that potentially contains a corresponding region of *q*. By this, we reduce the number of images to be considered and thus the computational effort considerably. Such a preselection is also conducted in the retrieval system of Seguin et al. [16, 18], where they first select the most promising images based on global image features so that a following sub-window search for localization becomes feasible. However, in contrast to our approach, such a holistic filtering is likely to miss small motifs which can not be recovered during the subsequent search.

In the second step, we focus on the most promising images with their local matches to predict the final retrieval bounding boxes. We consider only rectangles with identical aspect ratios and limit ourselves to position and scale changes, neglecting more complex transformations like rotation or shearing. By this, we do not have to increase the voting space and accelerate the search speed. Furthermore, the assumption of rectangular retrieval bounding boxes with identical aspect ratios is quite common and implicitly assumed in sliding window-based approaches [16, 17]. In the following, we denote **c**_*r*_ and *d*_*r*_ as the center and diagonal of an arbitrary rectangle *r*. Now, each local match (*f*, *g*) with g∈D(I) votes for a specific rectangle *r* in image *I*. We define **v**_*f*_ = **c**_*q*_ − **c**_*f*_ as the connection vector from the center of the local query patch **c**_*f*_ to the query rectangle **c**_*q*_. Then the local match (*f*, *g*) votes for a rectangle *r* in *I* with center **c**_*r*_ = **c**_*g*_+ **v**_*f*_ ⋅ *d*_*g*_/*d*_*f*_ and diagonal *d*_*r*_ = *d*_*q*_ ⋅ *d*_*g*_/*d*_*f*_. Since we presuppose identical aspect ratios as the query region, the retrieval rectangle is unambiguously defined by its center and diagonal. We fuse all those matches by creating a voting map similar to [54]. Therefore, the vote of local matches (*f*, *g*) are inserted into the voting map of image *I*, where each point of the voting map represents a retrieval box at the respective position. Hereby, the voting map is a down-scaled version of the image and we use the local similarity score defined in Eq 4 for the voting score. The voting map includes only the votes for retrieval bounding box centers. To estimate the scale, we average over the diagonals of the local matches voting for the same position. This way, we do not have to increase the voting space by another dimension and limit computational overhead. To reduce quantization errors and stabilize the voting, each local match votes for a small five by five window with a Gaussian kernel. Finally, we take the position of the maximum in the voting map and the averaged diagonal to obtain the center and diagonal of the best retrieval bounding box in image *I*. In contrast to [16, 17], our voting-based retrieval allows finding and localization at the same time without computational expensive sliding-window search over all image positions.

#### Local query expansion and re-voting (N3)

After the initial search, we integrate query expansion into our voting-based retrieval algorithm to refine search results further, see N3 in Fig 5. For this, the feature descriptors of the original query and highly-ranked retrievals are averaged. This often improves the search results since the best-ranked retrievals are typically correct and contain information not included in the original query. Moreover, by averaging the feature descriptors, a region is only ranked high if it has a small distance to the original query and all highly-ranked retrievals in the expansion. We use ten of its nearest neighbors for each local query patch and average their feature descriptors and apply L2 normalization. After expanding the local query patch descriptors, we also need to update the voting vectors due to changes in position and scale of the local matches due to the expansion. For this, we measure the voting vector from the expanded local query patch to the query region by finding the nearest neighbor of the expanded local query patch within the query image and measuring its offset vector and scale ratio concerning the query region. Finally, we repeat the voting procedure described previously and obtain refined retrieval results using the expanded local query patches and the updated voting vectors.

#### Interactive feedback

Our visual search interface allows users to give the system feedback to improve retrievals results further. After a search is finished and results are listed, users can mark retrievals as true and false and correct retrieval bounding boxes by adjusting their position. Based on this feedback, the search can be started again. Since it is hardly possible to update the feature representation with single results, we use the negatives and positives to train a linear support vector machine (SVM) to find a more discriminative search vector. Therefore, we extract feature vectors of true and false retrievals and sample in addition to the negatives a moderate number of feature vectors from random patches across the dataset analogously to the Exemplar SVM [75]. The random negative patches help to characterize the positives, especially if the users have marked very few negatives. The linear SVM is trained on this data, and we use the vector *w* that defines the orientation of the separating hyperplane as a new search vector across the index. Neglecting the bias term, the new search essentially classifies all regions whether they belong to the marked positives or not, with the nearest neighbors having the highest probability belonging to the positives. Based on this new search vector, we re-run the search similar to the local query expansion, which improves the search results since *w* was optimized to separate the positive from the negative retrievals.

#### Multi-region search

To allow art historians to analyze not only single but also compositions of several motifs, we allow the search for multiple regions in our interface, where users can determine the desired type and strength of the connection between regions he is looking for. Therefore, we proceed as follows. Let us consider the users marked *n* query regions *c* = (*q*_1_, …, *q*_*n*_) in image *I*_*q*_. Then we apply the single region search to obtain retrieval bounding boxes $R(q_{i})$ for each query region *q*_*i*_ across the whole image collection. Based on these retrievals for single regions, we try to find the best corresponding composition and arrangement of regions in other images. Therefore, we consider all possible retrieval combinations for each image and score them based on the desired connection type, where users can choose between an OR, AND, or geometrical connection between regions. Let us consider an image *I* with retrievals $R(I,q_{i})$ for *q*_*i*_ ∈ {*q*_1_, …, *q*_*n*_} and the similarity $s_{q_{i}}\left(r\right)$ for $r\in R(I,q_{i})$. Then we define the OR scoring function by

$$\begin{array}{c} S_{c}^{\text{OR}}\left(\left(r_{1},\ldots ,r_{n}\right)\right)=\text{max}(\{s_{q_{i}}\left(r_{i}\right)|\;i\in 1,\ldots ,n\}), \end{array}$$ (6)

where $r_{i}\in R\left(I,q_{i}\right)$ and we assume only one retrieval per query region for simplicity. If multiple retrievals for a query region in the image exist, we consider the most similar. This connection type prefers composition of retrieval regions with one region being as similar as possible to one of the query regions. For the AND scoring function, in contrast, we take the mean similarity over all regions, i.e.

$$\begin{array}{c} S_{c}^{\text{AND}}\left(\left(r_{1},\ldots ,r_{n}\right)\right)=\frac{1}{n}\sum_{i=1}^{n}s_{q_{i}}\left(r_{i}\right)), \end{array}$$ (7)

where $r_{i}\in R\left(I,q_{i}\right)$ and we consider again only one retrieval per query for simplicity. Therefore, it prefers composition of retrieval regions where all regions are as similar as possible to the query regions on average. Both connection types are independent of geometric relationships. However, for art history it is not only important whether specific motifs are present, but also how they are arranged. Therefore, we introduce a third function that also takes the geometric relationships into account and is inspired by classical objective functions for feature matching, see for example [64, 76]. It consists of two main parts, a unary and a binary term. Assuming that all regions of the query and retrieval regions are fully connected graphs, the unary term measures their similarities and the binary term how consistent all pairwise connection vectors are between the regions. For the binary term, we measure the deviation of relative distances between the regions and the deviation of the angle between the connection vector and the horizontal axis. Therefore, the scoring function consists of three terms, i.e.

$$\begin{array}{c} S_{c}^{\text{GEO}}\left(\left(r_{1},\ldots ,r_{n}\right)\right)=\underset{\text{unary}\;\text{term}}{\underset{︸}{\lambda_{s}\sum_{i=1}^{n}\phi_{q_{i}}^{s}\left(r_{i}\right)}}+\underset{\text{binary}\;\text{term}}{\underset{︸}{\lambda_{a}\sum_{i,j=1}^{n}\phi_{q_{i},q_{j}}^{d}\left(r_{i},r_{j}\right)+\lambda_{d}\sum_{i,j=1}^{n}\phi_{q_{i},q_{j}}^{a}\left(r_{i},r_{j}\right)}}, \end{array}$$ (8)

where $\phi_{q_{i}}^{s}\left(r_{i}\right)$ measures the similarity based on $s_{q_{i}}\left(r_{i}\right)$ and $\phi_{q_{i},q_{j}}^{d}\left(r_{i},r_{j}\right)$, $\phi_{q_{i},q_{j}}^{d}\left(r_{i},r_{j}\right)$ the agreement of relative distances and angles of the connection vectors $c_{q_{j}}-c_{q_{i}}$ and $c_{r_{j}}-c_{r_{i}}$, respectively. The parameters λ_*s*_, λ_*a*_ and λ_*d*_ are weighting individual terms against each other and can be adjusted by users according to their preferences before starting the search.

We consider the complexity of the multi-region search. The most time-consuming scoring is geometric scoring. Since we consider only the best retrievals per image and region, finding the optimal composition of regions scales cubically with the number of regions *n* which are searched for. However, in a typical application case, where we can assume *n* ≤ 5, the additional time spent searching for the other query regions takes much more time than evaluating the retrieval score. Therefore, since the independent search for regions increases linearly with *n*, the multi-region search with OR, AND, and geometric scoring also scales linearly with *n* in typical use cases.

## Art historical case study

Before we present comparative and diagnostic experiments of the retrieval algorithms on benchmarks, we demonstrate the usability of our interface for art historical research by providing an example of how the system is used in practice.

### Rubens’ artistic influence

Peter Paul Rubens (1577–1640) is one of the most important Dutch painters and his monumental history paintings and portraits are prime examples of Baroque art. His painterly style and chosen motifs and themes provided a source of inspiration for his fellow artists and future generations. Rubens is the starting point for our study, which examines Rubens’ influence, especially on artists belonging to his close circle, by taking the example of the horse. The motif is chosen because Rubens is considered to be a masterful painter of animals; especially horses achieved an unprecedented importance in his oeuvre. As a court painter to the governors of the Southern Netherlands Archduke Albert (1559–1621) and Archduchess Isabella (1566–1633), Rubens had access to their menagerie, which allowed him to study domestic and exotic animals in nature. He documented his observations in numerous, very detailed studies. His extensive travels to Italy and Spain and his knowledge of Renaissance bronze sculptures, especially equestrian statues, certainly also influenced his depictions of animals [77]. Thus our research question is as follows: To which extent was Rubens’ horse adapted by fellow artists? What can we say about the horse’s appearance (i.e. pose) and subject matters in which it is shown?

### Dataset and analysis

**Fig 7:**
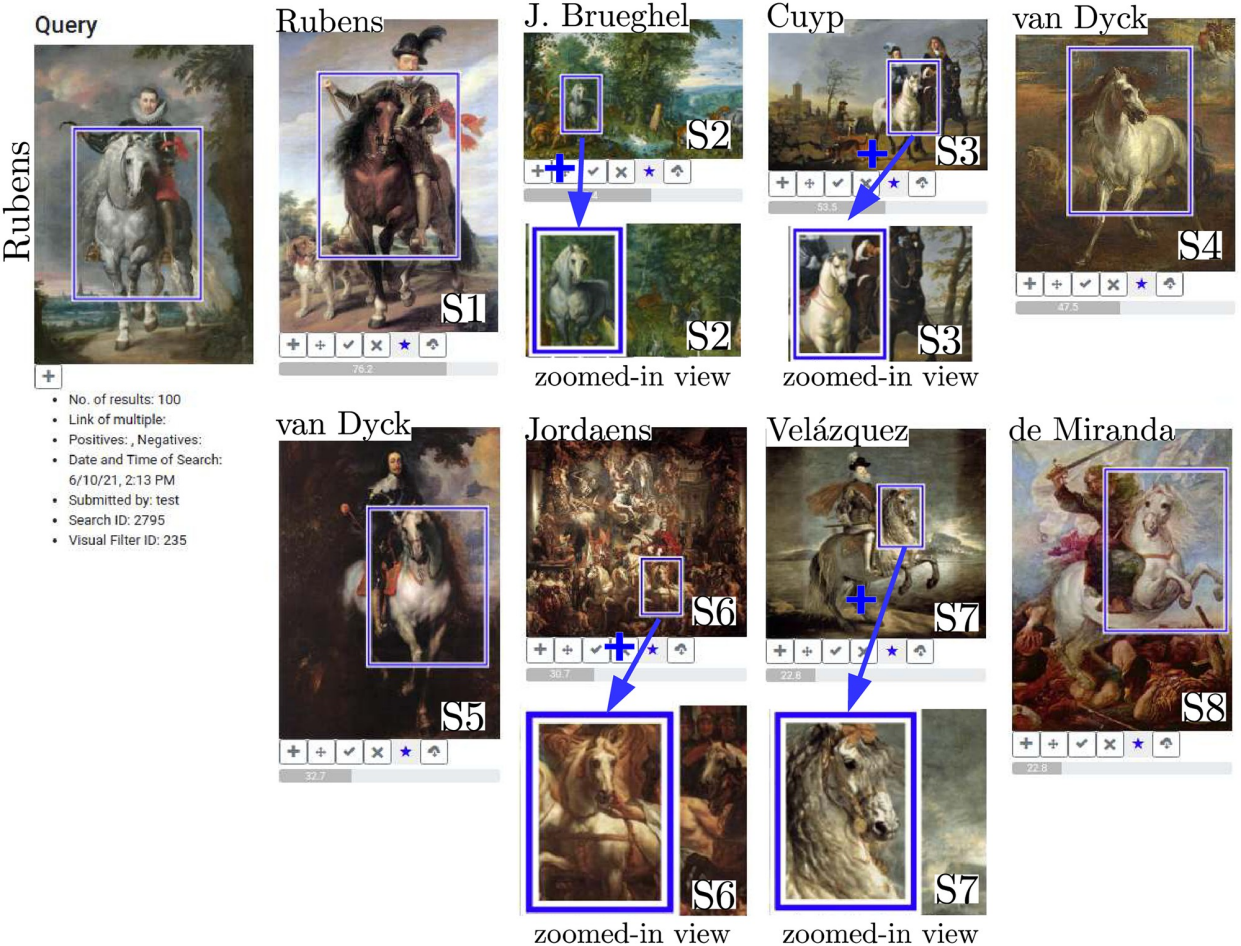
Search results for Rubens’ horse motif. Detail view of our interface showing the search for the motif of the horse in frontal view, a depiction popularized by Peter Paul Rubens. The selected query region is displayed on the left and retrievals marked as particularly interesting are displayed on the right. Favorites were selected from the first 40 results (first two retrieval pages). Additionally, we show the zoomed-in view given by our interface for some images and added the artist’s name. Image material is shared by Wikimedia Commons [5] either as public domain or under a CC0 license.

For this study, we have compiled a dataset of 12143 images from the Wikiart [6] and Brueghel [17, 78] dataset with additional Ruben’s paintings from Wikimedia Commons [5]. It contains images from the Renaissance period, especially High and Northern Renaissance, and Baroque. These periods were selected because they not only enclose Rubens artistic work but also enable us to identify and study potential preceding and succeeding examples. Between 1612 and 1615 Rubens painted a monumental portrait of Don Rodrigo Calderón (c.1580s-1621) on horseback. The size and painterly treatment suggest that the horse is of equal importance than the figure of Calderón, a Spanish ambassador in Antwerp. Rubens paints the white horse in frontal view, front and back legs are raised, thus indicating movement, its mane blowing in the wind. The head is slightly turned towards the right and depending on the hanging, its gaze is directed towards the viewer. It is a majestic and powerful representation of the horse. Later, art historians would write that Rubens “(…) popularized this type of authoritative representation [79].” To study Rubens’ influence we select the portrait of Calderón and define the horse as our search query. After the search has determined, results are displayed in another window; retrievals show that the interface detected the motif very confidently and frequently in the dataset. Looking at the results, it is noticeable that the animal appears at various locations in the image, in different sizes and poses, painted from various angles and embedded within diverse subject matters. The fact that the interface not only finds copies but also variations of a motif is valuable to art history, because it highlights stylistic developments and personal or cultural preferences. Fig 7 illustrates favorites from the retrievals which also visualize mentioned characteristics.

The first retrieval (S1) displays the horse in an identical manner to the query: in frontal view, the horse’s head is turned towards the right, legs are raised and its mane tousled by the wind. In fact only the color of the horse, now a dark brown tone, diverges from the query. The metadata reveals that the painting shows Sigismund III Vasa (1566-c.1632), king of Poland, and was executed by Rubens roughly ten years later, in circa 1624. Identical to the equestrian portrait of Calderón, the Dutch painter has chosen his prototypical horse to accompany a person of wealth and power. The horse has retained a similar appearance in Rubens’ equestrian portraits throughout the years. Stylistic and physical features reflect the royal qualities of the person depicted. This observation is supported by other retrievals from the first 40 results (first two retrieval pages) which include identical or very similar equestrian portraits by Rubens. These indicate that he had studied the subject intensively and eventually created a type, which he reverted to. S2 shows a painting by Jan Brueghel the Elder (1568–1625); if we use “J. Brueghel” in the following text study, we refer to Jan Brueghel the Elder and not to his son, Jan Brueghel the Younger (1601–1678). The horse has been detected on the left surrounded by a lush landscape and numerous domestic and exotic animals. The animal is shown very similar to Rubens’ horse, especially the pose, alignment and physical features resemble Rubens’ model. While J. Brueghel has created an almost identical copy he detached the motif from its historical context and integrated it into a new, biblical subject: the Paradise Garden. Thus we can establish a first link between the work of Rubens and J. Brueghel. If we consult the literature we find that both painters were not only bound by their profession or hometown of Antwerp but above all by their life-long friendship and partnership. Between 1598 and 1625 both painters collaborated on around twenty-four artworks, which united their distinct style [77]. Also J. Brueghel and Rubens visited each other in their studios, resulting in the fact that both borrowed motifs from each other [79].

**Fig 8:**
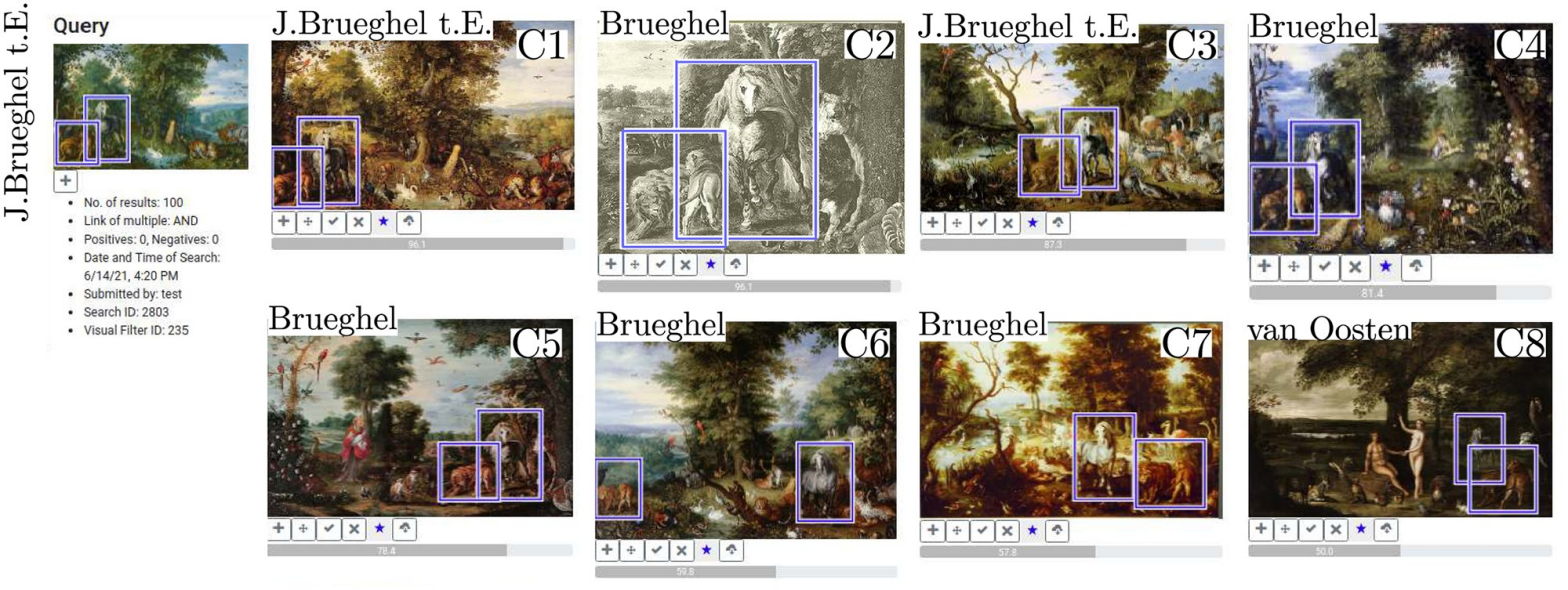
Search results for the horse and the group of lions. The selected query regions are displayed on the left, retrievals marked as particularly interesting are displayed on the right. Favourite retrievals were selected from the first 40 results (first two retrieval pages). Additionally, we added the artist’s name for a better orientation. “Brueghel” was used as an umbrella term and includes members of the Brueghel family, their workshops and successors who painted in their style. For these works, the attribution is uncertain. Image material is shared by Wikimedia Commons [5] either as public domain or under a CC0 license.

‘Paradise with Fall of Man’ (S2) was created by J. Brueghel in 1613 and displays a very intriguing ambiguity: it displays chaos and order at the same time. Especially the arrangement of horse and the group of growling lions is eye-catching. We make use of the fact that our interface enables to search for multiple image regions and study, if we find an identical or similar arrangement in other paintings. Thus we define both regions as queries. Again the interface detects the motif very confidently and based on the number of correct retrievals we can tell that it was indeed a very popular composition. By looking at the metadata we can make further statements about how and when the motif appears in other works of J. Brueghel and identify links to other artists. Before we interpret retrievals it must be added that precise attributions are difficult to make in the context of J. Brueghel. He belonged to a family of artists, who worked closely together, borrowed motifs and produced many copies of each others works. The Brueghel family includes numerous artists, however, most cited are Pieter Brueghel the Elder (c1525/30–1569), his sons Pieter Brueghel the Younger (1564–1636) and Jan Brueghel the Elder and the son of the latter, Jan Brueghel the Younger (1601–1678). Like his family, J. Brueghel operated a workshop and often it remains unclear if he or a member of his studio executed a work; also there are many successors who painted in the style of J. Brueghel. The following interpretation thus focuses on the composition and makes statements about artists only if possible. Fig 8 shows a selection of favorites from the first 40 results. C1 and C3 are attributed to J. Brueghel, painted in 1612 and 1613 respectively. Both retrievals show an identical arrangement to the query, where the lions are depicted to the left of the horse in close distance, seemingly unimpressed by each other’s company. While J. Brueghel painted the group at an identical position in C1, he moved their location to the center in C3. The appearance of the horse however remains identical to the query and to Rubens’ prototype. It is unknown if J. Brueghel himself, members of his workshop or successors produced the other paintings. It is noticeable however that in most retrievals the horse and the lions form a close group, i.e. C2 or C4. Only in some instances the group is separated–this can be seen in C6. This closeness might be due to the fact that both are royal symbols and in the case of J. Brueghel link to Archduke Albert and Archduchess Isabella, for whom he also served as a court painter [77]. His arrangement is also influenced by animal studies published in natural history books, which depict the lion first and the horse second [79]. Retrievals highlight that if changes were made then in relation to order, instead of the left, the lions appear on the right side of the horse. C8 is attributed to Dutch artist Izaak van Oosten (1613–1662) and was painted between 1651 and 1662, more than twenty years after J. Brueghel’s death. The retrieval suggests J. Brueghel’s influence and a popularity of the motif. Van Oosten adapted the horse and the group of lions, retaining a similar arrangement and appearance. Based on the retrievals we conclude that the horse and the group of lions was a popular motif in the work of J. Brueghel, his workshop and successors. Arrangement and appearance remain very similar over time, with only slight alterations. One most add that not only the appearance of the horse is influenced by Rubens but also the group of lions. His painting ‘Daniel in the Lion’s Den’ (circa 1614–1616) is often cited as a model [79]. It is noticeable that the arrangement mostly appears in paintings of biblical subjects. One might argue that by reusing it over time it became an established composition in Dutch art of the time to depict and identify biblical stories. This inserted study has demonstrated that our interface enables to search for specific arrangements and variations of it. It also highlighted that computer-based methods, in our case a user-interface, allow to follow a visual observation which emerged while answering a related research question.

We return to Fig 7. The motif of the horse is further detected in a painting (S3) by Albert Cuyp (1620–1691) entitled ‘Lady and Gentleman on Horseback’ (c.1655). Cuyp, a Dutch artist most known for his landscapes, creates a horse which appears much more calmer and less muscular than Rubens’ energetic prototype (S3). Its wildness and dynamic is replaced by a calmness and domesticity, reflected in the tamed mane. Also the perspective slightly changed: While Rubens presented a frontal view of the horse, Cuyp prefers a slightly shifted angle. Similar to Rubens, the horse is a trusty companion to the equestrian, but in Cuyp’s painting the Dutch landscape is protagonist. Eventually the horse looses its importance and presence to the surrounding landscape and figures. In S4 the horse is protagonist again and regained its wildness and energy. The artwork was painted between 1635 and 1645 by an unknown artist, who painted in the style of Anthony van Dyck (1599–1641). The influence of Rubens on its general appearance is less obvious, especially considering the chosen perspective, pose and less pronounced and imposing corporeality. Also the context of the motif has changed; in this particular image, the horse is no longer part of an equestrian portrait but claimed its own space. The link to Antwerp-born van Dyck is not singular, S5 shows a retrieval from an oil painting executed by van Dyck circa 1635–40. This time the reference to Rubens is clearly visible: Although his motif is mirrored, the horse is also shown in frontal view, with raised legs and in motion. The painting bears even more similarities regarding the structure, composition and genre. Like Rubens, van Dyck uses the horse in an equestrian portrait of King Charles I of England, thus retaining an identical context.

In the middle of the seventeenth century another Dutch artist adapted the motif, demonstrating Rubens lasting effect on younger generations of artists. Our interface detected the horse in an artwork (S6) by Jacob Jordaens (1593–1678), showing the triumph of Prince Frederik Hendrik (1584–1647). This retrieval is especially notable, because it highlights the ability of our interface to detect motifs in varying sizes and within crowded compositions. In the image, the detected horse is accompanied by three others all of which show strong similarities to Rubens’ model. So far all retrievals are assigned to Dutch artists, S7 and S8 diverge from this homogeneous group. In circa 1634/35, Spanish artist Diego Velázquez (1599–1660) painted an equestrian portrait of King Philip III of Spain (S7). His horse is shown from the side, with both forelegs raised but less energetic and impressive in its physical appearance. Rubens might have influenced Velázquez, especially considering that both have met during Rubens’ visit to Spain in 1628–29 [80], however, in this instance the appearance of the horse and its alignment in the image indicate stronger links to the tradition of equestrian sculptures of the Italian Renaissance. Velázquez fellow countryman Juan Juan Carreño de Miranda (1614–1685) placed the horse within a mythological context, as the companion of ‘Santiago in the Battle of Clavijo’ (1660). Although varying in pose, de Miranda’s horse displays a strength and energetic corporeality which establishes a link to Rubens’ prototype and highlights his lasting influence on later generations, even outside of the Netherlands.

### Findings

The previous section presented a case study which demonstrated how our interface is used in practice to address the following question: To which extent was Rubens’ horse adapted by fellow artists? What can we say about the horse’s appearance (i.e. pose) and subject matters in which it is shown? Based on an analysis of retrievals we can conclude the following: Rubens’ horse was not only reused by him many times–preferably in historical paintings or portraits–but also cited by numerous other artists. These belonged–as in the case of J. Brueghel–to his close circle or lived in the Netherlands. Eventually the retrievals of Jordaens (S6) or de Miranda (S8) highlighted that his influence surpassed time and space. It was noticeable that Dutch artists were often from Antwerp suggesting a tight network of Antwerp-based artists. This closeness and practice of artistic exchange manifested in paint: Notably the retrievals by van Dyck (S5) or J. Brueghel (S2) emphasized a strong connection to Rubens. Indeed it seemed that Rubens’ prototype, showing a strong and energetic animal, in frontal view with turned head was reused by artists. While its appearance changed slightly in some instances, for example in S4, S6 or S8, these character traits remained visible. We especially noticed a close link between J. Brueghel and Rubens. Retrievals showed that J. Brueghel adapted the horse, its appearance, including color and pose, closely resembling that of Rubens. Retrieval S2 illustrates this adaptation. The fact that the interface allows a search based on multiple image regions led us to perform a follow-up search, which was triggered by the peculiar arrangement of horse and a group of lions in the retrieval of J. Brueghel (S2). We asked if other paintings displayed an identical or similar arrangement. Retrievals showed that the arrangement was indeed very common in the works of J. Brughel, his workshop and successors but included variations regarding the order or distance between horse and lions.

Regarding the context we observed that the horse often appears in portraits but that it was also transferred into new contexts, namely biblical or mythological subjects. Our study showed that Rubens had a great influence on artists of his time but also beyond. While the horse is not a creation of his, it gained a muscular and energetic appearance and a strong presence in his paintings which was adapted by other artists. Eventually Rubens created a prototype which was distinct to him and therefore made his works highly recognizable.

## Experiments

This section presents ablation studies of various aspects of our multi-style feature aggregation and instance retrieval system and comparisons with state-of-the-art methods on benchmark datasets. In addition, we compare our system with existing visual search interfaces, including TinEye [22], Google [20] and Bing [21]. For implementation details of our algorithm, we refer to S1 File.

### Datasets and evaluations

Our evaluation is based on 5 challenging benchmarks including datasets of photos and artworks. We mainly follow Shen et al. [17] in our dataset selection, where we also consider two extensions to investigate the performance of our algorithm for heterogeneous and large scale datasets.

#### Brueghel

**Fig 9:**
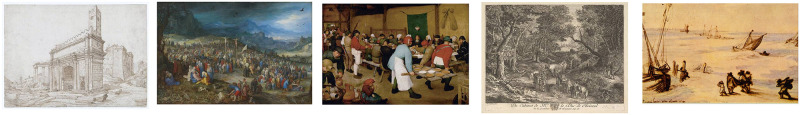
Examples of paintings in the Brueghel dataset. The depicted images show the diversity of artworks in the Brueghel dataset. It includes artworks of various subject matters (i.e. landscapes, genre paintings), in different techniques (i.e. drawing, oil, print) and materials (i.e. paper, canvas). Due to copyright issues, we exchanged several images. We made sure that replacements are as similar as possible to the originals. The image material is shared by Wikimedia Commons [5] as public domain.

The main evaluation is based on the Brueghel dataset by Shen et al. [17] with images from the Brueghel Family Database [78], for which they labeled several motifs with bounding boxes. To our knowledge, this is currently the only publicly available benchmark for finding and localizing similar and identical motifs in the arts. The dataset consists of 1587 paintings from the Brueghel family and their workshops, which are painted in different styles with changing artistic media and material. See Fig 9 for examples from the dataset. There are 10 different motifs labeled with 11 up to 57 instances across the dataset. We follow the evaluation of Shen et al. where retrievals are counted as correct if the intersection over union (IoU) is larger than 0.3. This threshold is rather small, but the authors justify the value as being sufficient for most retrieval applications. Based on the retrievals counted as correct and incorrect, the Average Precision (AP) is calculated for each query, averaged over the respective class, and afterwards averaged over all classes, yielding a mean Average Precision (mAP).

#### Brueghel5K & Brueghel101K

More than often, art historians are presented with extensive and heterogeneous art collections. To simulate this, we introduce two extensions of the Brueghel dataset, which we refer to as Brueghel5K and Brueghel101K. There we add 3,500 and 100,000 images as distractors to the dataset, respectively. The distractors are random images that have nothing to do with the query motifs and act as negatives. We sample distractors from the Wikiart dataset [6], where we excluded paintings by Peter Brueghel the Elder to circumvent that they contain a query motif by chance. The additional images include artworks from different artists, epochs, genres, and artistic styles resulting in a very heterogeneous dataset. The queries can now be mixed up with much more regions within the dataset, making the retrieval task much more difficult. For the evaluation on the two extensions, we proceed analogously to the previously described Brueghel dataset, where we also report the mAP as performance metric.

#### Large Time Lags Location (LTLL)

In addition to art datasets, we also evaluate our algorithm on photographs. Therefore, we test our algorithm on the Large Time Lags Location (LTLL) dataset from Fernando et al. [81]. It consists of 225 historical and 275 modern photographs of 25 different landmarks. The images are taken at different periods which are spanning over 150 years. The dataset was introduced to recognize the location of an old image given the locations of the modern photographs. This can be understood as a retrieval task, i.e. given the historical photograph to find the most similar modern photograph and to assign the respective location. The main challenge of the dataset is to bridge the gap between different domains. Historical and modern photographs differ in terms of their visual appearance (i.e. color, perspective, contrasts, texture) and therefore can be considered as two different domains. Our retrieval system acts on the assumption that users only mark image regions they are interested in. Therefore, we provide and utilize annotated query bounding boxes for our and all baseline models. Following the evaluation protocol of Fernando et al. [81] and in the spirit of a 1-nearest neighbor classifier, we report the accuracy of the first retrieval.

#### Oxford5K

We also evaluate our approach on the Oxford5K buildings dataset from Philbin et al. [56]. The dataset is one of the most commonly used benchmarks for instance retrieval and consists of 5,062 high-resolution photos comprising 11 different Oxford landmarks, including towers, cathedrals, and bridges. The images were taken from Flickr with respective search terms for the landmarks and additional distractors. For each landmark, there are 5 queries with 7 up to 220 occurrences. We follow the evaluation protocol of [56] and compute the Average Precision (AP) for each query, average all APs per landmark and report the mean Average Precision (mAP).

### Effect of the multi-style feature aggregation

First, we validate the performance improvements using the multi-style feature aggregation. For this, we investigate the performance changes of our retrieval algorithm on all datasets for different feature descriptors. As baselines, we use ImageNet and Artminer features. Therefore, we use a VGG-16 with batch normalization [72] which is truncated after the conv-4 layer and either pre-trained on ImageNet only (ImageNet) or additionally fine-tuned with Artminer’s self-supervised training on the respective dataset afterwards (Artminer). The results are reported in Table 1.

**Table 1:** Comparison of feature representations and fusion strategies.

Features	Brueghel			LTLL	Oxford5K
	[17, 78]	5K	101K		
ImageNet	82.0	78.3	70.2	87.6	89.5
Artminer [17]	83.4	39.9	38.5	89.5	80.4
Ours w/max	87.2	83.7	76.2	92.3	90.7
Ours w/concat	86.4	84.0	**79.3**	90.7	**91.2**
Ours	**87.5**	**84.3**	77.3	**92.6**	**91.2**

Performance comparison of the retrieval algorithm with the multi-style features (Ours) against pre-trained ImageNet (ImageNet) and fine-tuned Artminer (Artminer) features. Additionally, we compare different aggregation strategies, including taking the max (w/max), concatenating (w/concat), and averaging over the stylizations.

Observing the results, we note that our approach improves retrieval results for all datasets. In particular the performance gain for art datasets is significantly high compared to the initial model. This is to be expected, because art datasets contain images with varying subject matters, styles or techniques and thus include large domain shifts. For the Oxford5K dataset we note a rather small improvement. We also achieve better results using the multi-style features in comparison to the self-supervised approach of Artminer. This is particularly large for the very heterogeneous datasets of Brueghel5K and Brueghel101K, where we obtain twice as high mAP scores. This also holds for the Oxford5k dataset, which contains many distractors and relatively few repetitions for their training and where our performance is significantly higher. For most datasets the analysis of fusion strategies shows that taking the average gives superior or comparable results. Only for Brueghel101K, we observe that the concatenation leads to a slightly better retrieval performance.

### Effect of the number of style-templates

We examine the influence of the number of style templates used for the multi-style feature aggregation. We first create different sets of style templates by grouping the target feature space of *ϕ*_*s*_ into different numbers of clusters *k*_*s*_ and extract their templates as described previously. Then, the performance of the retrieval algorithm for these style templates with different *k*_*s*_ is measured and reported in Table 2.

**Table 2:** Effect of the number of style templates.

k_s_	Brueghel	LTLL	Oxford5K
1	86.3	91.4	91.0
2	87.2	91.2	**91.3**
3	**87.5**	**92.6**	91.2
4	86.5	91.1	90.3
5	85.9	90.2	90.6
6	85.7	91.1	91.2

We measure the performance of the retrieval algorithm for searching with different numbers of style templates *k*_*s*_ in the multi-style feature aggregation.

From the table, we can observe that we already get a performance improvement for *k*_*s*_ = 1 compared to the initial feature descriptors. Thus, merging with one stylization already reduces the domain-induced differences and improves the retrieval performance. Regarding the Brueghel dataset, it can be seen that the performance first increases with the number of style templates, but then after *k*_*s*_ = 3 it decreases continuously. We assume that by aggregating an increasing number of stylizations, more and more fine-grained information is lost, leading to a decrease in retrieval performance beyond a certain point. This effect is probably more pronounced for the Brueghel dataset compared to the LTLL and Oxford5K datasets because there the motifs are much smaller. In average over all datasets, we obtain the best performance for *k*_*s*_ = 3.

### Effect of the multi-scale region-of-interest pooling

To study the effect of the multi-scale region-of-interest pooling, we reduce the number of scales in the region pooling and investigate its influence on the retrieval performance of our retrieval system with the multi-style aggregated features. Results can be found in Table 3.

**Table 3:** Effect of the multi-scale region-of-interest pooling.

#scales	Brueghel	LTLL	Oxford5K
1	86.3	90.0	85.8
3	85.9	91.2	90.5
5	86.8	**92.6**	89.9
7	**87.5**	**92.6**	**91.2**

We measure the performance of the retrieval algorithm for searching with different numbers of scales in the multi-scale region-of-interest pooling approach.

We observe that the reduction of scales has a direct impact on the performance. If we do not use a multi-scale approach, the performance for all datasets deteriorates significantly. This has a particularly large influence on the Oxford5K dataset, where the performance drops by more than 5 per cent in mAP. This is not surprising since scale differences are most significant in this particular dataset.

### Effect of the iterative voting

We investigate the influence of iterative voting on the performance of the retrieval algorithm. Therefore, we compare our whole retrieval system against searching for the entire query region without voting (wo/voting) and searching without the local query expansion and re-voting (wo/re-voting). Additionally, we consider the performance on the Brueghel dataset with different IoU thresholds. This allows us to analyze the influence of voting on the localization more precisely. In general, the higher the threshold, the more precisely the retrieval bounding boxes have to match the ground truth boxes to be considered a correct retrieval, and the worse the overall mAP values are. The results are reported in Table 4.

**Table 4:** Effect of the iterative voting.

Methods	Brueghel			LTLL	Oxford5K
	IoU@0.3	IoU@0.5	IoU@0.7		
Ours wo/voting	75.9	55.7	7.1	74.6	70.7
Ours wo/re-voting	76.0	59.9	28.0	91.2	89.3
Ours	**87.5**	**67.8**	**31.2**	**92.6**	**91.2**

We measure the performance of the retrieval algorithm for searching only with the entire query region (wo/voting), restricting ourselves to the first round of voting (wo/re-voting) and the entire approach. Additionally, we report results on the Brueghel dataset for different IoU thresholds.

Results show that iterative voting significantly improves the performance for all datasets. In particular, we note that results increase after the first round of voting for the LTLL and Oxford5K datasets. Considering the Brueghel dataset, the first round of voting enhances finding additional instances only slightly. However, the localization improves notably, which is reflected in the performance gain for higher IoU values. Further improvements are made by the local query expansion and re-voting. Compared to the Brueghel dataset, this effect is much smaller for the LTLL and the Oxford5K datasets. Reason being that the Brueghel dataset contains very similar, almost identical, instances of several motifs. This is visible in the search results, where the first retrievals are very similar to the given query and thus are very suitable for query expansion. However, our ablation study also shows that even without the local query expansion and re-voting procedure, our results are already better or at least comparable to the state-of-the-art on the Brueghel dataset [17].

### Effect of the local patch selection

**Fig 10:**
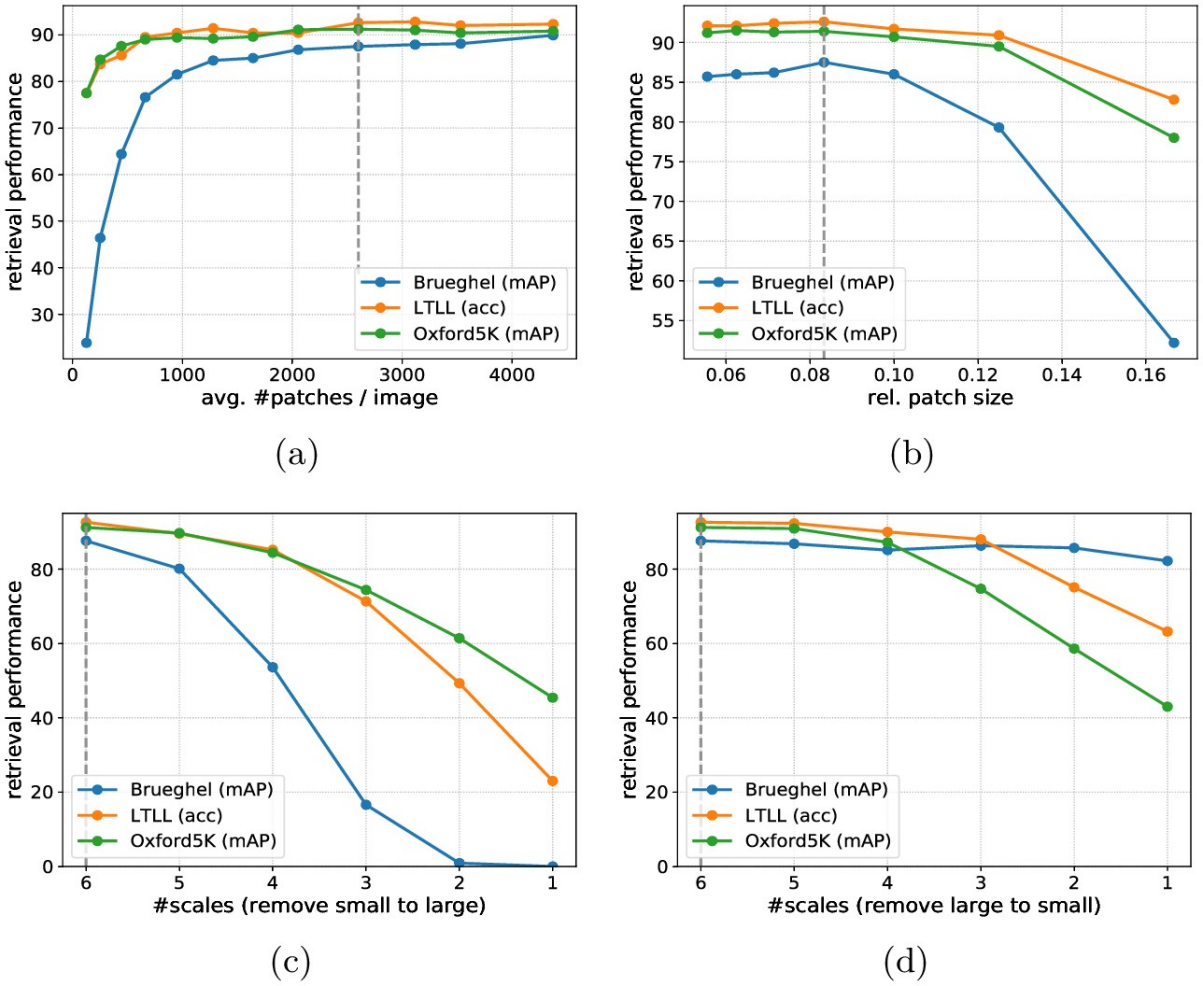
Effect of the local patch selection. Retrieval performance was measured for different average numbers per image (a), different sizes (b), and covered scales (c,d) for the patches on different datasets. We have successively reduced the number of scales starting with the smallest (c) and starting with the largest (d) local patches. We have marked our default configuration with a dashed line in each plot.

In the following, we consider the influence of the local patch selection on our retrieval algorithm. For this purpose, we analyze the dependency of the retrieval performance on the average number per image, the size, and the covered scales of the local patches on different datasets. First, we vary the step size of the sliding window from 1/65 to 1/10 of the largest image side for the smallest scale, which directly affects the average number of patches per image, see Fig 10a. Then, to investigate the dependency on the size we vary the patch size starting with 1/18 up to 1/6 of the largest image side for the smallest scale, see Fig 10b. Finally, to investigate the influence of the scales, we successively decrease them and start with the smallest or largest scale, see Fig 10c or 10d, respectively.

Considering the average number of local patches per image, see Fig 10a, we observe that the performance first increases quickly and then starts to saturate. A sufficient number seems to be especially important for the Brueghel dataset since it contains very small motifs, and thus a good coverage is essential for accurate localization. Any value over 2000 patches per image provides good results for the considered datasets. Only the Brueghel dataset seems to benefit from even more local patches, but an expanding search index is a significant drawback to this. Regarding the local patch size, see Fig 10b, we can observe a similar behavior. Starting with rather large local patches, the performance improves with decreasing patch size and then begins to saturate or slightly worsens. This is plausible since the patches are not yet too small that they do not contain enough image information, and the voting approach allows searches based on these small patches to be fused into a meaningful retrieval. On the other hand, if the patches are too large, no local patches can be found within the query region, and the co-occurrences of the queries in the dataset cannot be adequately covered by these patches, which particularly manifests for the Brueghel dataset. The results concerning scale changes, see Fig 10c and 10d, show that small scales have a significantly larger influence. This is especially true for the Brueghel dataset, as it contains much smaller motifs. However, including higher scales has a positive influence on the performance of the LTLL and especially the Oxford5K dataset.

### Computational cost

In the following, we study the search speed and report on the memory consumption of the search index in more detail. Since the search index has to be stored on the GPU for optimal speed, its size is a potentially limiting factor. We compare our algorithm with the approach of Shen et al. [17]. For a fair and meaningful comparison, all experiments are conducted on the same machine with three GPUs (NVIDIA Quadro P5000). For [17], we divide their search times on a single GPU by three to account for the possibility to distribute their search on three GPUs. We also report the search times for running our algorithm on the CPU. In Table 5, we provide a summary of all results for a single query and the memory consumption on the GPU for varying dataset sizes.

**Table 5:** Comparison of search speed and index size.

Method	CPU/GPU	5K	20K	40K	60K	80K	100K
Artminer [17]	GPU	12.9 min	50.4 min	1.7 h	2.8 h	3.8 h	4.6 h
Ours	CPU	6.6 s	8.8 s	9.5 s	10.6 s	12.0 s	13.5 s
Ours	GPU	**6.5 s**	**7.0 s**	**7.7 s**	**8.4 s**	**8.7 s**	**8.9 s**
Ours	GPU	3.5 GB (1)	6.3 GB (1)	17.7 GB (2)	20.4 GB (2)	23.4 GB (2)	27.3 GB (3)

Search speed comparison between our algorithm (Ours) and [17] (Artminer) for varying dataset sizes (upper part). Additionally, we report the used memory on the GPU for the nearest neighbor search (lower part). Numbers in brackets indicate how many GPUs were used to store the index.

The table shows that our search algorithm needs about 9 seconds to process a dataset containing around 100k images. This is much faster than the sliding-window based approach of [17], although they have parallelized their search as a convolution on the GPU. Furthermore, we observe that the search time only moderately depends on the dataset size. We examine a large offset in the search time due to the multi-scale feature extraction of the search region, which can be further optimized in the future. A limiting factor of the number of images that can be searched is the index size. The major factor that determines its size is the number of local patches extracted per image. Essentially this number influences how small the query regions can be, while still be found reliably. Since search requirements and available hardware depend on the application, the number of local patches should be adjusted to the individual use case. Another possibility to decrease the index size is to reduce the dimension of the local patch descriptors. However, our experiments showed that the retrieval performance significantly decreases for feature dimensions smaller than 96. We also observed that the nearest neighbor search on CPU is a valid option, which decreases the search time only by few seconds. And for smaller datasets, this has no influence at all.

### Benchmark performance

In the following, we provide quantitative and qualitative experiments of our retrieval algorithm on the previously presented benchmarks.

#### Quantitative evaluation

We compare the performance of our retrieval algorithm against competitive baselines. This includes max-pooled features of a CNN pre-trained on ImageNet (ImageNet, max-pool) and the state-of-the-art approaches of Shen et al. [17] (Artminer) and Radenović et al. [15]. For all methods, we use a VGG-16 with batch normalization as backbone architecture and also report the performance of [17] with their proposed ResNet-18 model. In contrast to [17], we utilize annotated query regions for the LTLL and Oxford5k dataset. We also evaluated their algorithm with query regions, however, they did not profit and obtained better results with their so-called discovery mode using full images as queries. This allows their algorithm to consider and utilize more context information and leads to overall better retrieval results. Therefore, we report the results of their so-called discovery mode. The retrieval performances of all methods on all datasets are summarized in Table 6.

**Table 6:** Comparison of retrieval performances on different benchmarks.

Methods	Net.	F.-tuned	Dim.	Brueghel			LTLL	Oxford5K
				[17, 78]	5K	101K		
ImageNet, max-pool	VGG-16	no	512	24.0	22.5	17.7	47.8	25.6
Radenović et al. [15]	VGG-16	no	512	15.5	12.7	5.9	59.3	53.4
Radenović et al. [15]	VGG-16	yes	512	15.8	12.8	5.7	76.1	87.8
Artminer [17]	ResNet-18	no	256	58.1	56.0	50.2	78.9	84.9
Artminer [17]	ResNet-18	yes	256	76.4	46.5	37.4	88.5	85.7
Artminer [17]	VGG-16	no	512	54.4	50.5	44.1	81.8	85.0
Artminer [17]	VGG-16	yes	512	79.9	39.5	36.4	88.9	81.5
Ours	VGG-16	no	64	84.3	80.3	69.5	90.9	89.4
Ours (default)	VGG-16	no	96	87.5	84.3	77.3	92.6	91.2
Ours	VGG-16	no	128	87.8	86.2	78.9	92.6	**91.4**
Ours	VGG-16	no	256	**89.0**	**86.9**	**80.3**	**93.1**	91.1

The retrieval performance of our method (Ours), max-pooled features, and state-of-the-art methods on benchmark datasets. We also provide information on the network architecture (Net.), whether or not features are fine-tuned on the retrieval task (F.-tuned), and the utilized feature dimensions (Dim).

The table shows that we outperform all baselines on all benchmarks. This is remarkable since we do not fine-tune the image representation on the retrieval task, and we rely on a much smaller feature dimension. Nevertheless, we are able to combine many weak search queries with the voting procedure to reliable search results. It can also be observed that our approach is much more stable for heterogeneous datasets with a lot of distractors so that the performance only moderately decreases for Brueghel5K and Brueghel101K. In contrast, [17] shows already a large performance drop on the Brueghel5K dataset compared to the initial network before the fine-tuning. The reason is that their self-supervised approach depends on finding corresponding regions. However, with many distractors the probability is very high that for a randomly selected region, either no or a wrong correspondence is found and thus either too few or wrong training examples are generated. Concerning the Oxford5K dataset, we see that we even outperform Radenovic et al., although their approach was explicitly trained for landmark localization on a large landmark dataset in a self-supervised manner. Their approach performs extremely poorly on the art datasets, which is not surprising since, on the one hand, their features are fine-tuned for landmark localization, and on the other hand, they train a global image representation. However, such a global image representation is not suitable to find a diverse set of small motifs in other images reliably.

#### Qualitative evaluation

**Fig 11:**
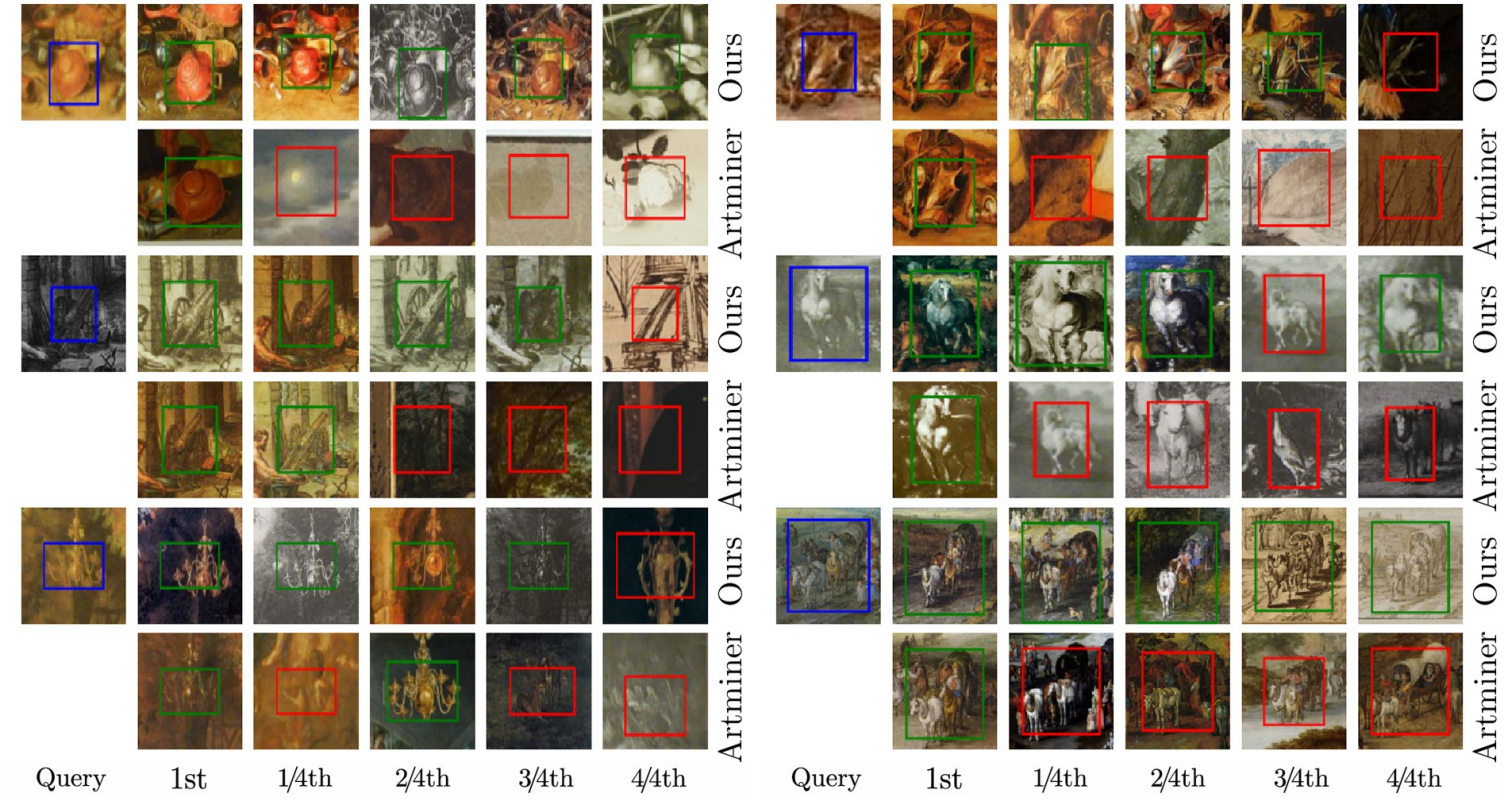
Qualitative comparison of our method with Artminer. Search examples of our approach (Ours) and Shen et al. [17] (Artminer) on the Brueghel dataset. Queries are shown in blue on the left and retrievals on the right. If the IoU is greater or smaller than 0.3, we draw green or red bounding boxes, respectively. For a better overview, we draw only the first and four additional retrievals with equidistant ranks. We set the distance between ranks to the number of ground truth matches of this query divided by four. Due to copyright issues, we replaced several images. We made sure that replacements are as similar as possible to the originals. The original figure is also available on our project website. The image material is shared by Wikimedia Commons [5] as public domain.

In the following, we provide a qualitative comparison on the Brueghel dataset against the state-of-the-art [17] (Artminer) and additional qualitative examples of our search system on all benchmarks. For a better overview we only show a few retrievals per query at certain rankings. The visualized retrievals have been evenly distributed over the total number of annotated instances. In Fig 11 we present a direct comparison of our method with Artminer for different queries and ranks. For both algorithms, retrievals in the first few ranks are correct. As we approach higher ranks, we note that our method finds significantly more instances.

**Fig 12:**
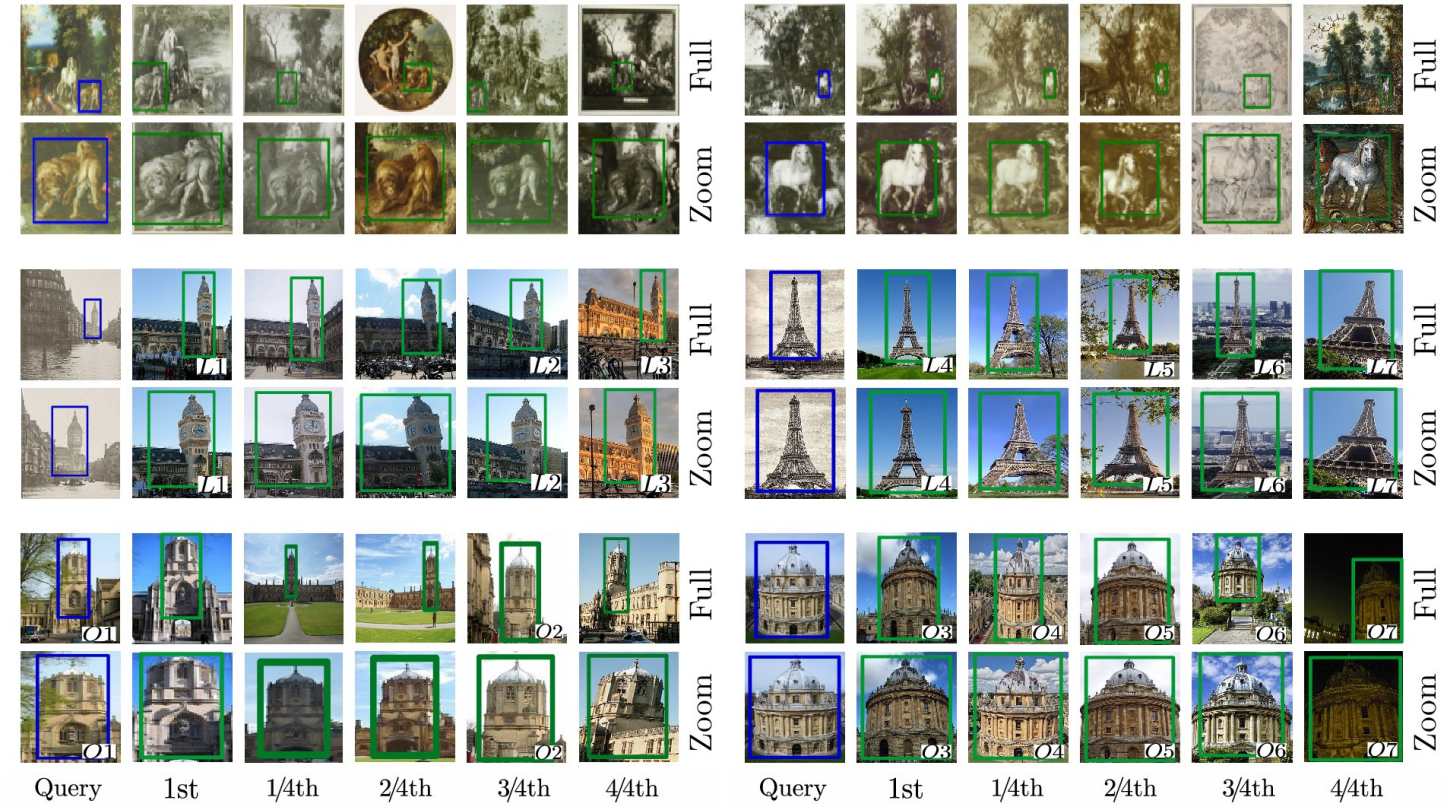
Retrieval examples on Brueghel, LTLL and Oxford5K. Rows 1–2, 3–4 and 5–6 show search examples of the Brueghel, LTLL and Oxford5K dataset, respectively. To allow for a better comparison, the first rows show retrievals in the full image (Full) and the second rows display enlarged versions (Zoom). Queries are shown in blue on the left and retrievals on the right. We highlight correct and incorrect retrievals with green and red bounding boxes, respectively. For a better overview, we draw only the first and four additional search results with equidistant ranks. We set the distance between between ranks to the number of ground truth matches of the query divided by four. Due to copyright issues, we replaced several images. We made sure that replacements are as similar as possible to the originals. The original figure is also available on our project website. Image (L4), (L7), (O1), (O2), (O4), (O6) and (O7) are slight modifications of [82–88] licensed under CC BY 2.0. Image (L1), (L2), (L3), (L5), (L6), (O3) and (O5) are slight modifications of [89–95] licensed under CC BY 3.0. The remaining image material is shared by Yale University Art Gallery [96] or Wikimedia Commons [5] either as public domain or under a CC0 license.

In Fig 12, we provide qualitative retrieval examples on Brueghel, LTLL, and Oxford5K. We show retrievals on both the entire image and in close-up to allow a detailed comparison of the query and retrievals. Search results demonstrate that our algorithm can find similar objects despite differences in color or other style features. Furthermore, we observe that even small objects are localized precisely despite changes in scale, color, perspective and illumination.

### Qualitative comparison with visual search interfaces

**Fig 13:**
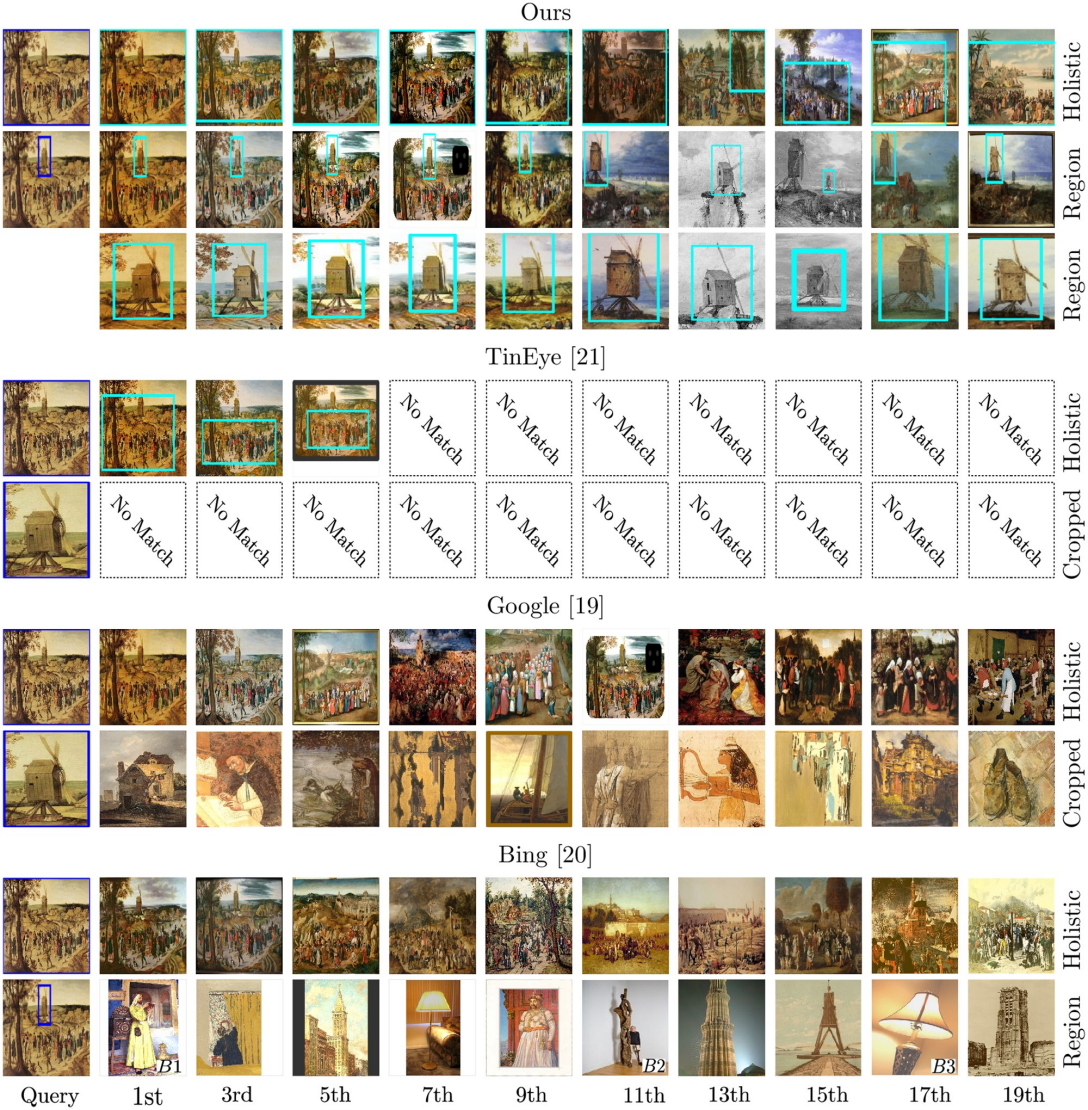
Qualitative comparison with TinEye, Google and Bing image search. We show retrieval examples for a given query on a modified Brueghel dataset for our algorithm and TinEye [22] and on the web for Google [20] and Bing [21] image search, where we made sure that all algorithms can find all visualized retrievals. We show results for a holistic image search (Holistic) as well as on the cropped (Cropped) and marked region (Region). In addition, we visualize retrievals in full screen (first row) and zoomed-in version (second row) for our results of the regional search. Due to copyright issues, we replaced several images. We made sure that the replacements are as similar as possible to the originals. The original figure is also available on our project website. Image (B1) and (B2) are slight modifications of [97, 98] licensed under CC BY 3.0. Image (B3) is a slight modification of [99] licensed under CC BY 2.0. The remaining image material is shared by Wikimedia Commons [5] either as public domain or under a CC0 license.

A quantitative comparison with existing visual search interfaces is difficult since underlying methods are often not published [20–22, 24], or they are only provided for specific datasets and the code is not available [18, 23]. In the following, we make a qualitative comparison with search systems which are available to us. This includes Google [20], Bing [21] and TinEye [22]. These systems allow searching across the web [20, 21] or individual image collections [22] given a visual query. For the comparison, we proceed as follows. We select a random query region from the Brueghel dataset [17, 78] and search for most similar images using Bing’s and Google’s image search. Here we only considered retrievals indexed by both algorithms, i.e. we check whether we can find respective retrievals with both algorithms if we search for them. We integrate these retrievals into the Brueghel dataset and eliminate repetitions. In this modified Brueghel dataset, we search with TinEye and our algorithm. Again we only consider retrievals which are indexed by Google and Bing. Therefore, we make sure that all algorithms can find all presented retrievals. We perform a holistic and regional search for all systems as far as possible. For TinEye and Google, it is not possible to select a region for the search. Thus, we crop the desired region and perform a holistic search on the cropped region. The results are visualized in Fig 13, where we only show every second retrieval to cover a larger search range.

The following observations can be made. First, TinEye is not able to find semantically similar images. There must be some identical parts in both images to identify the images as a match. Therefore, their algorithm can only find slightly alternated duplicates, and thus the number of retrieved images is very limited. Furthermore, the algorithm does not find almost identical images since it is very sensitive towards changes. Their algorithm also retrieve the most similar region within the retrieved image to the query image, visualized by the cyan bounding boxes. The search with the cropped region shows that the search is not regional. Although an almost identical copy of the windmill is depicted in some images, the motif is not found. Since there is no close-up of the same windmill in the dataset, TinEye does not obtain any search result. If we turn towards the holistic search results of Google and Bing, we observe that first search results are reasonable. However, the weakness of the holistic image search already becomes evident in these results. The search is greatly influenced by the people in the foreground and the color composition of the image, regardless of the actual region the user is interested in. A more constraint search for individual motifs is not possible. Again a region-based search does not improve the performance. Although Bing offers the possibility to select single regions, a comparison to the holistic search shows that the algorithm searches for similar images to the cropped region and not for similar regions within other images. Although many similar images with identical objects are in their index (see their holistic search), they are not retrieved because images are holistically encoded on the dataset side. Overall we conclude that in contrast to our approach, existing visual search systems do not meet the requirements of art historians and hence can not be used in practice. Reasons being that they are very susceptible to color and do not allow to search for regions containing specific objects and motifs in other images. Eventually these system were not built for art historical research.

## Conclusion and future work

In this article, we have presented an instance retrieval algorithm and a comprehensive user interface to find and localize similar or identical regions in an extensive digital art collection. Analyzing these visual relationships allow art historians to identify relations between artist, study the popularity of motifs or to examine how they have been adapted and altered in form and content over space and time. For this, we present a specifically tailored image representation for the arts. The introduced multi-style feature aggregation maps all images into the same distribution to reduce their domain-specific differences and improve the retrieval results across art collections. Thereby, we do not need strong supervision in terms of labeled image pairs or curated datasets for self-supervised training compared to previous approaches. The actual retrieval algorithm is based on iterative voting in combination with fast nearest neighbor search, where we fuse several weak sub-region searches into a reliable final search result. Compared to holistic based approaches, this allows us to find various small motifs reliably within images across an extensive dataset. We have shown that our approach significantly outperforms current state-of-the-art for visual instance retrieval in the arts in terms of accuracy and search time. Furthermore, we have demonstrated that our method applies to real-world scenarios with large and heterogeneous image collections. In addition to the search algorithm, we have developed a user interface that allows art historians to use our algorithm in practice. The interface holds numerous useful features, for example being able to select favorites, searching for single and multiple regions and to refine search results based on user feedback.

We have also demonstrated the usability and implications in the context of an art historical research question. Thereby, the study has amplified the following implications. First, retrievals have demonstrated the high-performance of computer-based methods and accuracy of results. The fact that our interface finds identical and similar motifs and compositions of multiple regions is valuable for art history and allows to ask complex research questions relating to content, stylistic developments or personal and cultural preferences. Our study of the horse motif has demonstrated that by utilizing the interface, we were able to study Rubens’ artistic influence and to identify artistic networks. Also, observing slight variations due to, for example, a change in subject matter or personal preferences of the artist. Moreover, our interface enables an easy access to image collections and an explorative and intuitive analysis, which is especially valuable for art historians who are less familiar with a period or an artist’s oeuvre. Lastly, we infer from our study of the arrangement of horse and lions that retrievals provide sources and starting points for new and complementary research questions which can be addressed instantly and quickly.

In future work, we want to accelerate our search further by using the MapReduce design pattern when a compute cluster is available. This should allow scaling the dataset size almost arbitrarily with the number of available working machines. A further extension of the interface offers the localization of corresponding areas beyond the commonly used rectangular bounding boxes. Although the major challenge for art historians is to find similar motifs and regions in tens of thousands of images, a pixel-wise localization and highlighting of corresponding areas between the query and retrievals would be convenient. Therefore, a subsequent co-segmentation or registration algorithm on the retrieval bounding boxes would be a nice extension of the interface.

## Supporting information

S1 File PDF file with implementation details. In this PDF file we give further implementation details of our algorithm.
(PDF)

S1 Video Demonstration of the visual search interface. The purpose of the video is to illustrate the functionalities of the user interface. During the review process the interface is accessible as an online web application on our project website https://compvis.github.io/visual-search. For login please use the user credentials provided on the project website and follow the steps described in the video. If the visual search interface is not reachable or other technical issues occur, please contact the corresponding authors.
(MP4)

S1 Data Additional data. The majority of utilized benchmarks are publicly available. To complete the data, we provide download links of the Wikiart distractors used to create the Brueghel5K and Brueghel101K datasets. Furthermore, we provide download links and labels of the Wikiart images used for training the style classification network and the additional query bounding boxes of the LTLL dataset. The data is available under https://compvis.github.io/visual-search.
(ZIP)
