# Supplementary material for: Large-scale interactive retrieval in art collections using multi-style feature aggregation
Source: PLoS One. 2021 Nov 24;16(11):e0259718. doi: 10.1371/journal.pone.0259718 (PMC8612525; doi:10.1371/journal.pone.0259718)
Supplement: S1 File — In this PDF file we give further implementation details of our algorithm. (PDF) [file pone.0259718.s001.pdf]

Supporting information: Large-scale interactive retrieval in art collections using multi-style feature aggregation

Implementation details

In the following, we give further implementation details to our algorithm. The implementation is based on Python 3.6, Pytorch 1.2 [1] and Faiss 1.6 [2], where the experiments were conducted on NVIDIA Quadro P5000 graphic cards.

Multi-style feature fusion

We use VGG-16 [3] with batch normalization as backbone architecture, pre-trained on ImageNet [4], and truncated after the fourth convolutional block. We rescale all images to 640 pixels concerning the smallest image side for feature extraction and padded them by 40 pixels on each side, which helps to find objects near image boarders. For the style transfer, we use the model of Li et al. [5]. The encoder consists of the first layers of the VGG-19 model [3], pre-trained on ImageNet [4], and the decoder of its symmetrical counterpart. It is trained on MS-COCO [6] and the weights are fixed for the remaining training. The rest of the network is trained on MS-COCO [6] and Wikiart [7] as content and style dataset for the style-transfer, respectively. We also experimented with models trained on each retrieval dataset separately, but this did not improve the performance and significantly increased computational complexity of the offline preparation stage. For more details of the style transformation model, we refer to [5]. To speed up the search, we stylize all images during the offline preparation. After averaging the feature maps of all stylized images, we apply a max-pooling to obtain a stride of 16 between image and feature space. For the extraction of local patch descriptors, we used Precise ROI Pooling [8] with a five by five grid, resulting in a feature dimension of 12800 for each local patch descriptor. Compared to other pooling methods, we obtained slightly better results using this pooling layer. After the pooling, we perform L2-normalizing on the features and apply principal-component analysis and whitening (PCAW) to reduce their feature dimension to 96. The PCAW is learned on a set of random local patch descriptors, which we generate by randomly selecting 500 images and 100 patches for each image. For the multi-scale region-of-interest pooling we use 7 different scales with two octaves and three scales per octave. Given a patch, we select the smallest scale of the feature map so that the corresponding area in the feature map has at least the size of the ROI Pooling grid.

Retrieval system using iterative voting

Our sliding-window approach for the local patch descriptors uses six different scales with a scaling factor of $2^{-1/2}$. The patch size ranges from 1/12 up to 1/2 of the largest image side, where we use a stride of 1/50. To keep the search index compact, we extract at most 4000 local patches and reduce the maximal number by 375 for each additional 20K images, starting with a dataset size of 20K. Concerning the search index, the OPQ algorithm utilizes 96 sub-quantizers with 8 bits allocated for each sub-quantizer. For the IVF algorithm, we generate 1024 clusters, where we use 30 for the nearest neighbor search. We train the OPQ and IVF algorithm on a set of random local patch

descriptors, which we generate by randomly selecting 1000 images and a maximum of 6000 patches for each image. For the local query expansion it is necessary to save all descriptors of the search index on disk since its is not possible to reconstruct them from the index. Therefore, we train a second product quantizer with the same configuration and store the PQ codes in an HDF5 file in the same order. For the query expansion the PQ codes are loaded and the feature vectors are reconstructed based on the centroids. This minimizes the memory size of the features on disk and we are be able to restore them very quickly during the online search. Concerning selecting most discriminative local query patches within the query region, we proceed as follows. For all local query patches, we remove all patches which have less than 95 percentage coverage and an intersection over union larger than 0.1 with the query region. On the remaining patches we apply non-maximum suppression on the feature activation to obtain the 25 most discriminative patches. For the voting map we keep the aspect ratio of the image and set the largest side to 100 pixels.

References

1. Paszke A, Gross S, Massa F, Lerer A, Bradbury J, Chanan G, et al. PyTorch: An Imperative Style, High-Performance Deep Learning Library. In: Wallach H, Larochelle H, Beygelzimer A, d'Alché-Buc F, Fox E, Garnett R, editors. Advances in Neural Information Processing Systems 32. Curran Associates, Inc.; 2019. p. 8024–8035.
2. Johnson J, Douze M, Jégou H. Billion-scale similarity search with GPUs. arXiv preprint arXiv:170208734. 2017;.
3. Simonyan K, Zisserman A. Very deep convolutional networks for large-scale image recognition. arXiv preprint arXiv:14091556. 2014;.
4. Deng J, Dong W, Socher R, Li LJ, Li K, Fei-Fei L. Imagenet: A large-scale hierarchical image database. In: 2009 IEEE conference on computer vision and pattern recognition. Ieee; 2009. p. 248–255.
5. Li X, Liu S, Kautz J, Yang MH. Learning linear transformations for fast arbitrary style transfer. arXiv preprint arXiv:180804537. 2018;.
6. Lin TY, Maire M, Belongie S, Hays J, Perona P, Ramanan D, et al. Microsoft coco: Common objects in context. In: European conference on computer vision. Springer; 2014. p. 740–755.
7. Wikiart;. `https://www.wikiart.org`.
8. Jiang B, Luo R, Mao J, Xiao T, Jiang Y. Acquisition of localization confidence for accurate object detection. In: Proceedings of the European Conference on Computer Vision (ECCV); 2018. p. 784–799.
